# Genetic and phenotypic differences between sexes in congenital hypogonadotropic hypogonadism (CHH): Large cohort analysis from a single tertiary centre

**DOI:** 10.3389/fendo.2022.965074

**Published:** 2022-12-02

**Authors:** Silvia Federici, Biagio Cangiano, Giovanni Goggi, Dario Messetti, Elisabetta Veronica Munari, Myriam Amer, Luca Giovanelli, Faris Hrvat, Valeria Vezzoli, Luca Persani, Marco Bonomi

**Affiliations:** ^1^ Dept. of Medical Biotechnology and Translational Medicine, University of Milan, Milan, Italy; ^2^ Dept. of Endocrine and Metabolic Diseases and Lab. of Endocrine and Metabolic Research, IRCCS Istituto Auxologico Italiano, Milan, Italy

**Keywords:** congenital hypogonadotropic hypogonadism (CHH), genetics, female, GnRH (gonadotropin releasing hormone), phenotype [mesh], sex

## Abstract

**Background:**

Congenital hypogonadotropic hypogonadism (CHH) is a condition with a strong genetic background, caused by a deficient production, secretion, or action of gonadotropin-releasing hormone (GnRH). Published data on CHH cohorts indicate a male predominance, although this is not supported by our current understandings.

**Aims:**

In order to unravel the possible causes or contributors to such epidemiological sex difference, the aim of our study is to investigate differences in genetic background and clinical presentation between males and females in a large cohort of CHH patients.

**Materials and methods:**

We enrolled 338 CHH patients with absent or arrested pubertal development, referred to our Center from 01/2016. Data collection included clinical assessment at diagnosis and genetic analysis performed by next generation sequencing (NGS), employing a custom panel of 28 candidate genes.

**Results:**

Among 338 patients 94 were female (F) and 244 male (M), with a ratio of 1:2.6. We found that 36.09% (122/338) of patients harbored potentially pathogenic rare genetic variants (RVs) with no significant differences between sexes; on the other hand, a significantly higher frequency of oligogenicity was observed in females (F 9,57% 9/94 vs M 3,69% 9/244, *P =* 0.034). The prevalence of non-reproductive phenotypic features was significantly higher (*P =* 0.01) in males (53/228, 23.2%) than in females (10/93, 10.8%): in particular, kidney abnormalities affected only male patients and midline defects had a significantly higher prevalence in males (*P =* 0.010). Finally, BMI SDS was -0.04 ± 1.09 in females and 0.69 ± 1.51 in males, with a statistically significant difference between groups (*P =* <0.001).

**Conclusion:**

Our data confirm the male predominance in CHH and identify some differences with regard to the clinical presentation between males and females that could indicate a variable expression of genetic rare variants and a dimorphic modulation of phenotype according to metabolic/behavioral factors, which will need to be substantiated and investigated by further studies.

## Introduction

**Figure 1:**
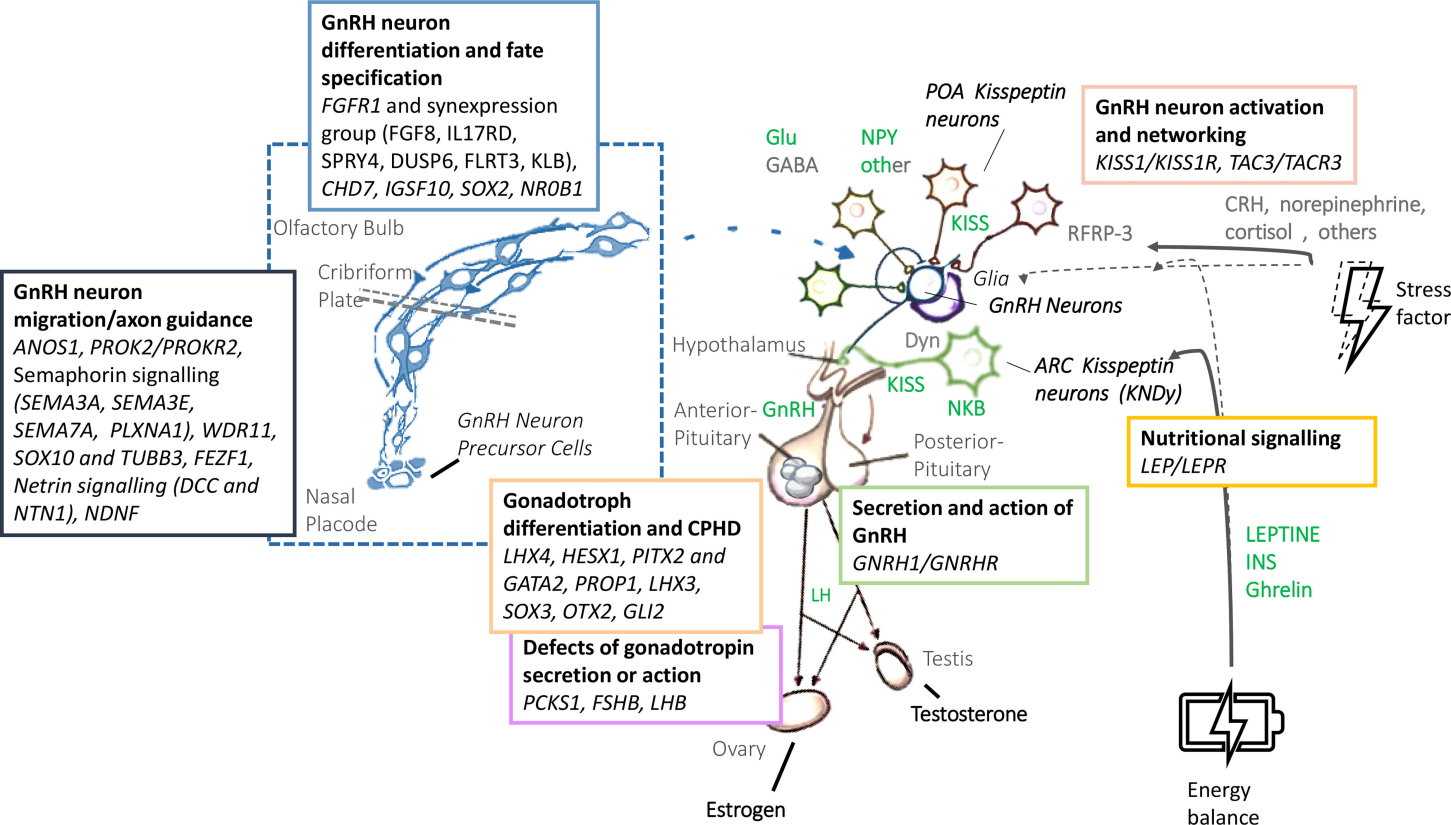
Biology and genetic background of the GnRH neuronal system. GnRH, gonadotropin-releasing hormone; LH, Luteinizing Hormone; FSH: Follicle-Stimulating Hormone; Glu, glutamate; GABA, gamma-amino-butyric acid; NPY, neuropeptide Y; POA, preoptic area; KISS, Kisspeptin; NKB, neurokinin B; Dyn, dynorphin; CRH, cortitropin releasing hormone; ARC, arcuate nucleus; INS, insulin.

Human pubertal development and reproduction is under the control of the hypothalamus-pituitary-gonadal axis, whose master regulator is represented by the GnRH-secreting neurons. Regular functioning of these neurons is the result of a sophisticated, complex and interconnected network which integrates different central and peripheral signals (**Figure 1**) (1). A failure in the correct development and/or activation of the human GnRH-secreting neurons lead to a congenital GnRH-deficiency named Congenital Hypogonadotropic Hypogonadism (CHH). CHH is a rare and complex disease characterized by a GnRH deficient production, secretion, or action. Despite its pathogenesis has not been completely understood yet, several evidence from familial pedigrees and animal models suggest a strong genetic background. Currently, more than sixty genes have been associated with CHH; nonetheless, as much as 50% of cases remain without an identified genetic cause (2). CHH can be clinically associated or not with a defective sense of smell, identifying, respectively, Kallmann syndrome (KS) and normosmic CHH (nCHH). Once accounted as two entirely separate diseases, KS and nCHH are now largely considered as different manifestations of the same genetic disease, since they often coexist in the same kindreds and they partly share the same genetic milieu (3). With the exception of ANOS1 mutations in hemizygosity, which are almost invariably associated with a defective sense of smell, mutations of genes involved in GnRH neuron migration, neuron fate specification or differentiation have been associated with variable degrees of olfactory defects, with either KS or nCHH phenotypes. Instead, rare variants of CHH genes associated with impairment of GnRH neuron activation or action are responsible for nCHH only (4).

Over the last fifty years, different studies have screened an ever-increasing number of candidate genes for CHH thanks to the use of Next Generation Sequencing (NGS) techniques, demonstrating an escalating impact of oligogenicity, which contributes to explaining the apparent variable penetrance and expressivity of some variants. However, since NGS allows the simultaneous screening of a wide number of genes, it also identifies a large number of rare variants of uncertain clinical significance (VUS). As pointed out in a recent survey among Expert Centers of the European Reference Network for rare endocrine conditions (ENDO-ERN) (5), the challenge is therefore to identify truly pathogenic variants more reliably, and distinguish true oligogenic inheritance from incidental rare findings that are not related to CHH.

The true prevalence of CHH is limited by the scarcity of published literature. In the original study the prevalence was estimated in 1:4,415 males (6), whilst more recently a Finnish retrospective study described an incidence of KS of 1:48,000, 1:30,000 in males and 1:125,000 females (7).

Indeed, CHH is traditionally considered a male predominant condition, with a male-to-female ratio of 4-5:1 (8–10), although when familial cases were analyzed separately, the ratio dropped to 2.3:1 (10). More recently there has been a reassessment of such imbalance, with the latest studies conducted among large patient cohorts reporting a male to female ratio of 3.6:1 (11) and 2.7:1 (12) and the sex ratio of affected individuals is closer to be equal in CHH kindreds (13, 14).

Nonetheless, the epidemiological sex imbalance, although apparently less consistent than previously considered, is not supported by the current understanding of CHH genetic basis. Indeed, only 3.5–10% (7, 15, 16) of cases harbor *ANOS1* mutations with a recognized X-linked inheritance.

The aim of this study is therefore to investigate possible differences in genetic background and clinical presentation in a large cohort of CHH patients, to unravel the possible causes or contributors to the observed epidemiological difference between males and females. An analysis of the female population will also be conducted to shed light on genotypic and phenotypic features.

## Materials and methods

### Study Population

We evaluated 338 CHH patients (94 females and 244 males, age at diagnosis 16.86 ± 3.11) who were referred to our Academic Medical Centre to perform genetic investigations and were therefore consecutively recruited from January 2016 to December 2021. Relevant patients’ data were retrospectively assembled as part of routine clinical practice based on the delivery of good clinical care, accomplishing the Declaration of Helsinki. The study was approved by the Ethic Committee of the coordinating institution (GR-2008-1137632), and all patients (or their parents/guardians) gave a written informed consent. All subjects were affected with pre-pubertal CHH, defined as the lack of complete spontaneous pubertal development. CHH was diagnosed as (1): manifestations of hypogonadism and delay/arrest of puberty associated with low testosterone/estradiol and inappropriately low/normal gonadotropins (2); absence of any known acquired cause of hypogonadotropic hypogonadism (i.e., expansive hypothalamic/pituitary lesions, hemochromatosis, etc.), or multiple pituitary hormone defects (MPHD). In order to remove the functional hypothalamic defects, the exclusion criteria were: (I) severe weight loss or eating disorder (17); (II) intensive exercise (>12 hours/week); (III) chronic illness and psychiatric disorders. Both patients with either a normal sense of smell or olfactory defects (hypo- or anosmia), as demonstrated using Brief Smell Identification Test (B-SIT) and/or MRI, were included. Anonymous patients’ data at the time of diagnosis, before starting any hormonal treatment, were retrospectively collected and a clinical database was created. Each patient’s genetics and disease phenotype were also reported.

### Phenotypic characterization

The age at diagnosis and the presence of any reproductive and/or non-reproductive phenotypical feature associated with CHH (the so called “red flags”: anosmia, cryptorchidism, microphallus, deafness, kidney abnormalities, midline defects and bimanual synkinesis) were recorded for each patient. The presence of anosmia allowed for a diagnosis of KS, and was considered separately from the other features. The presence of family history (defined as a history of pubertal delay, confirmed secondary hypogonadism in the absence of other obvious causes, or reproductive and nonreproductive defects associated with CHH in relatives up to the second degree) was also recorded. In addition, the segregation pattern of rare variants was recorded, whenever available. Moreover, anthropometric parameters (height, weight, and body mass index (BMI) were recorded in 50/94 (53.19%) females and 116/244 (47.54%) males. Standard deviation scores (SDS) according to the WHO age curves were obtained using Growth Calculator 3.0, in order to compare different reference standards between sexes. In addition, Tanner stages (18) at diagnosis for mammary development (Breast Tanner Stage) and pubic hair (Pubic hair Tanner Stage) in females, and for genitalia and pubic hair in males, were evaluated. Finally, uterine length (as in the longitudinal diameter of the uterus assessed on pelvic ultrasonography) was measured in females. All data were collected prior to any hormonal treatment.

For the female population we recorded the hormonal investigations carried out at diagnosis: basal LH, FSH and estradiol (17βE_2_) determination and dynamic testing with GnRH analogue. In particular, the LHRH stimulation test was performed using a standard protocol that involves taking basal venous blood samples for FSH and LH (0’), the subsequent intravenous administration of LHRH Ferring 0.1mg/1ml 100 µg, and the collection of blood samples at 30’, 60’, 90’ and 120 minutes for FSH and LH.

Due to the retrospective design of this study and the need to consider all values at diagnosis, different methods of hormonal measurement have been used. Nonetheless, in most cases, serum LH, FSH and 17βEstradiol concentrations were measured by electrochemiluminescence immunoassay “ECLIA”. These LH and FSH assays have a lower limit of detection of 0.1 IU/L, while estradiol assays usually have a lower limit of detection of 5 pg/mL. For the purposes of statistical analysis, LH and FSH values below the lower reference limit were estimated as 0.1 U/L, while 17βE2 values below the lower reference limit were estimated as 5 pg/ml.

### Genetic analyses by targeted next generation sequencing (NGS)

Each patient underwent a genetic investigation, using a targeted NGS technique, to look for rare allelic variants. We extracted the genomic DNA of each patient from peripheral blood lymphocytes using Gene Catcher gDNA 96 × 10 mL Automated Blood kit (Invitrogen, Life TechnologiesTM, Carlsbad, CA, USA). The CHH gene panel was designed using Illumina Design Studio (San Diego, CA, USA) and included the following CHH candidate genes: *ANOS1, FGFR1, PROKR2, PROK2, GNRHR, GNRH1, GNRH2, KISS1, KISS1R, TAC3, TACR3, HS6ST1, FGF8, CHD7, DUSP6, FEZF1, FGF17, FLTR3, IL17, SEMA3A, SEMA3E, SEMA7A, SOX2, SOX10, SPRY4, WDR11, HESX1, NELF*. The 28 CHH genes, consistently represented in all sequence capture panels, were assessed for the purposes of this study. Libraries were prepared using Illumina Nextera Rapid Capture Custom Enrichment kits according to the manufacturer’s protocols. All regions not correctly sequenced were recovered with NexteraVR DNA Library Preparation kit (Illumina, San Diego, CA, USA). For subsequent analyses, we included as “rare variants” (RVs) all known pathogenic, rare non-synonymous or splicing-site variants (Minor Allele Frequency, MAF ≤ 0.01) and novel non-synonymous or splicing-site variants. The frequency and the functional annotation of the identified variants were checked in public and licensed databases (Ensembl, UCSC Genome browser, 1000 Genome project, ExAC Browser, NCBI, HGMD professional), considering the ethnic groups (Europeans). We excluded common non-synonymous variants with Minor Allele Frequency (MAF) >0.01, synonymous, intronic, and 5′ or 3′ UTR variants. Each variant found was confirmed by Sanger direct sequencing using BigDyeVR Terminator v.3.1 Cycle Sequencing Kit (Life Technologies, Carlsbad, CA, USA) on a 3100 DNA Analyzer from Applied Biosystems (Foster City, CA, USA). In order to check for pathogenicity prediction, VarSome database (19) was used (up to October 2022): only the RVs classified as likely pathogenic, pathogenic, or variants of uncertain significance (VUS), according to the American College of Medical Genetics (ACMG) classification guidelines (20), were considered for further analysis.

### Statistical methods

Statistical analysis was performed using SPSS statistical package, version 27.0 (SPSS Inc., Chicago, IL, USA). Genetic and phenotypic variables were compared between the male and female populations. Moreover, comparisons were made according to the diagnosis (either KS or nCHH), the presence of any “red flag” at clinical presentation, and the enrichment in rare genetic variants at genetic investigation. Finally, in the female population comparisons were made according to Tanner stages. Either χ^2^ or Fischer’s exact test was used to compare categorical variables between groups. Comparisons for continuous variables were performed using independent samples t tests (for parametric data) and independent samples Mann-Whitney test or Kruskal Wallis test (for nonparametric data). Data are expressed as mean ± SE unless otherwise indicated. A p-value <0.05 was considered statistically significant.

## Results

Among our 338 patients there were 94 females (F) and 244 males (M), with a female to male ratio of 1:2.6; 147/338 patients (43.5%) had a diagnosis of KS and 191/338 (56.5%) of nCHH, with no significant differences in their prevalence between the two sexes.

### Sex difference in genetic background

In the whole cohort we identified a total of 245 rare variants, which were classified according to the American College of Medical Genetics and Genomics and Association for Molecular Pathology guidelines (ACMG/AMP) (20): 18.78% (46/245) resulted to be benign, 18.78% (46/245) likely benign, 24.49% (60/245) variants of uncertain significance, 24.49% (60/245) likely pathogenic and 13.47% (33/245) pathogenic (**Supplementary Tables 1, 2**). Benign and likely benign variants were excluded from further statistical analysis.

**Figure 2:**
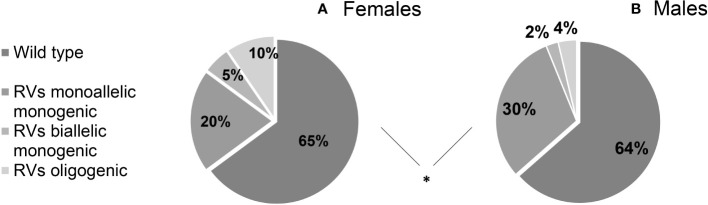
Prevalence of rare genetic variants in female **(A)** and male **(B)** cohort of patients according to their genetic assortment. RVs, rare genetic variants. Comparison between male and female patients using Fisher’s exact test: *P = <0.05.

We found that 36.09% (122/338) of patients harbored potentially pathogenic rare genetic variants (RVs), with no significant differences between sexes (F 35,11% vs M 36.48%). RVs were monogenic and monoallelic in 27.51% (93/338) of patients (F 10/94, 20.21% vs M 74/244, 30.33%), monogenic and biallelic (RVs in homozygosis) in 3.25% (11/338) (F 5/94, 5,32% vs M 6/244, 2,46%) and finally, there was an oligogenicity in 5.33% (18/338) of cases (F 9/94, 9,57% vs M 9/244, 3,69%). The genetic assortment of RVs was significantly different between females and males (**Figure 2**), with oligogenic and biallelic variants found more frequently in females (*P =* 0.034). This difference is maintained even after excluding patients with *ANOS1* RVs (*P =* 0.036).

**Figure 3:**
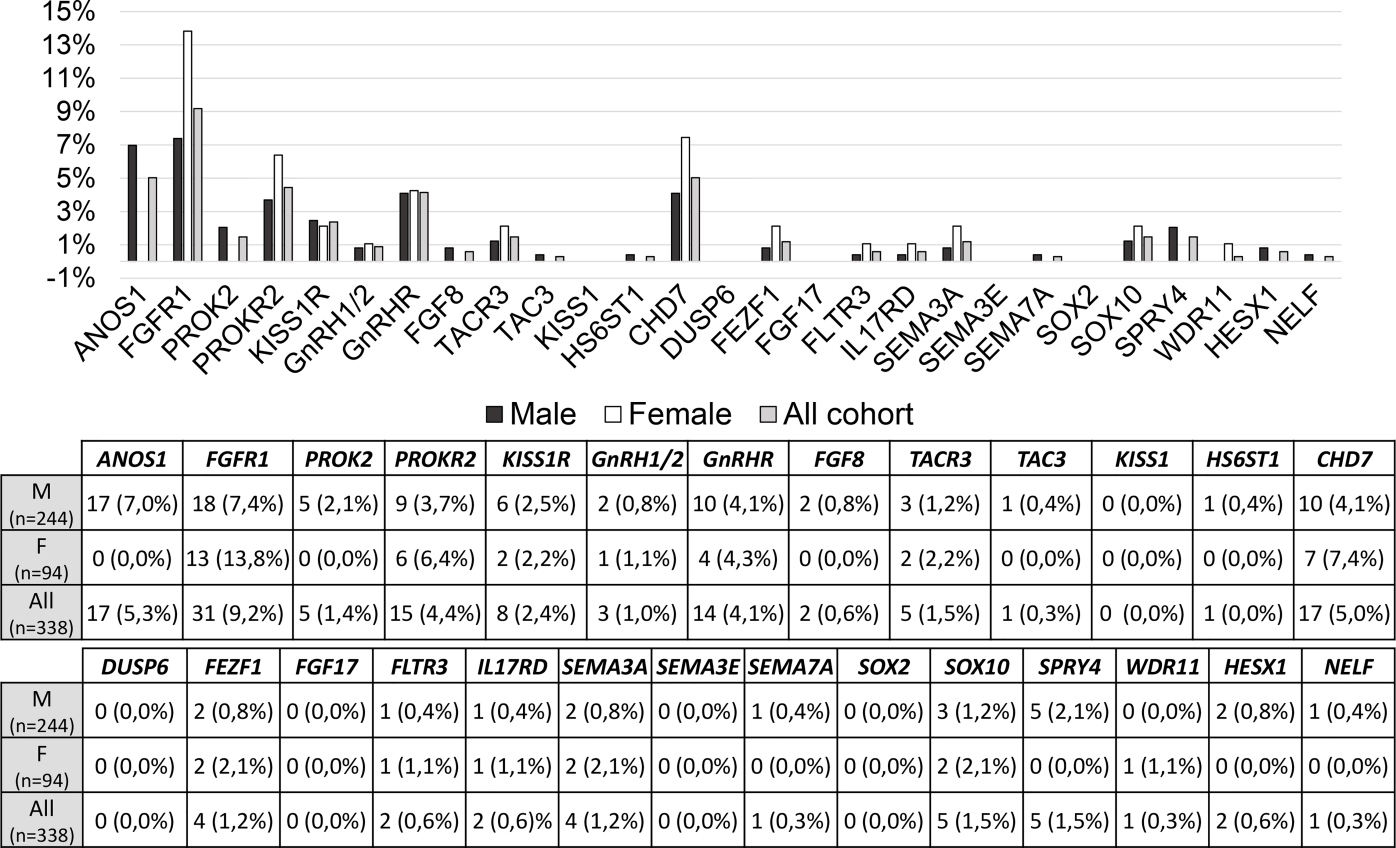
Prevalence of rare genetic variants in each candidate gene. RVs, rare genetic variants.

The prevalence of rare variants in each candidate gene is shown in **Figure 3** and no significant differences were found between males and females; however, RVs within *ANOS1* were found only in males, as expected.

### Sex difference in clinical presentation

Family history of pubertal delay or hypogonadism was found in 104/332 patients (31.3%): 35/92 (38.04%) females and 69/240 (28.75%) males (*P =* 0.246). No statistically significant differences were found in the prevalence of family history according to neither the diagnosis (KS vs nCHH) nor the enrichment in rare CHH genetic variants.

**Figure 4:**
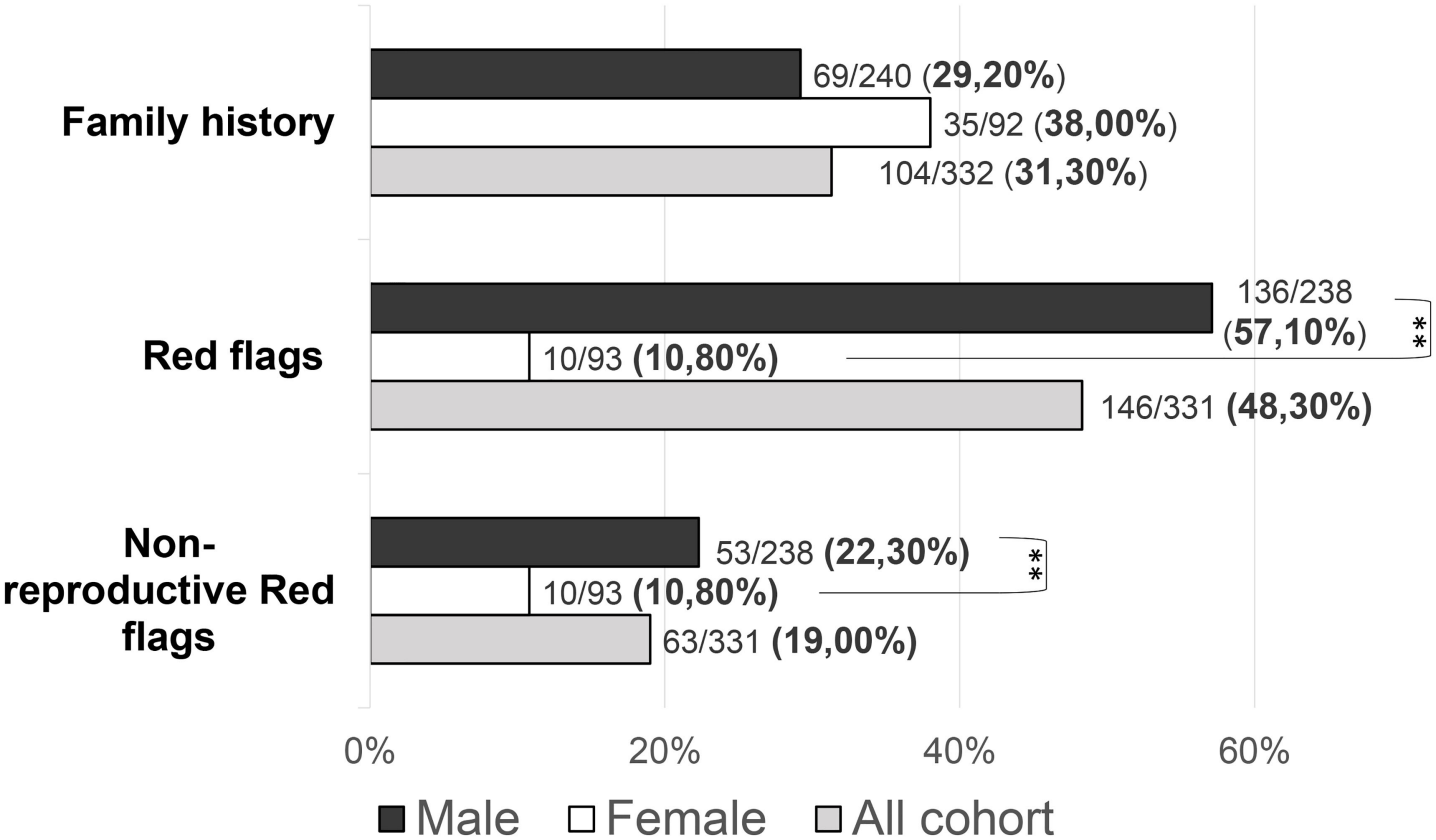
Prevalence of “red flags” and each “non-reproductive” defects in the study cohort Red flags: cryptorchidism, microphallus, deafness, kidney abnormalities, midline defects and bimanual synkinesis; Non-reproductive red flags: deafness, kidney abnormalities, midline defects and bimanual synkinesis. Comparison between male and female patients using Fisher’s exact test: **P = <0.01.

**Figure 5:**
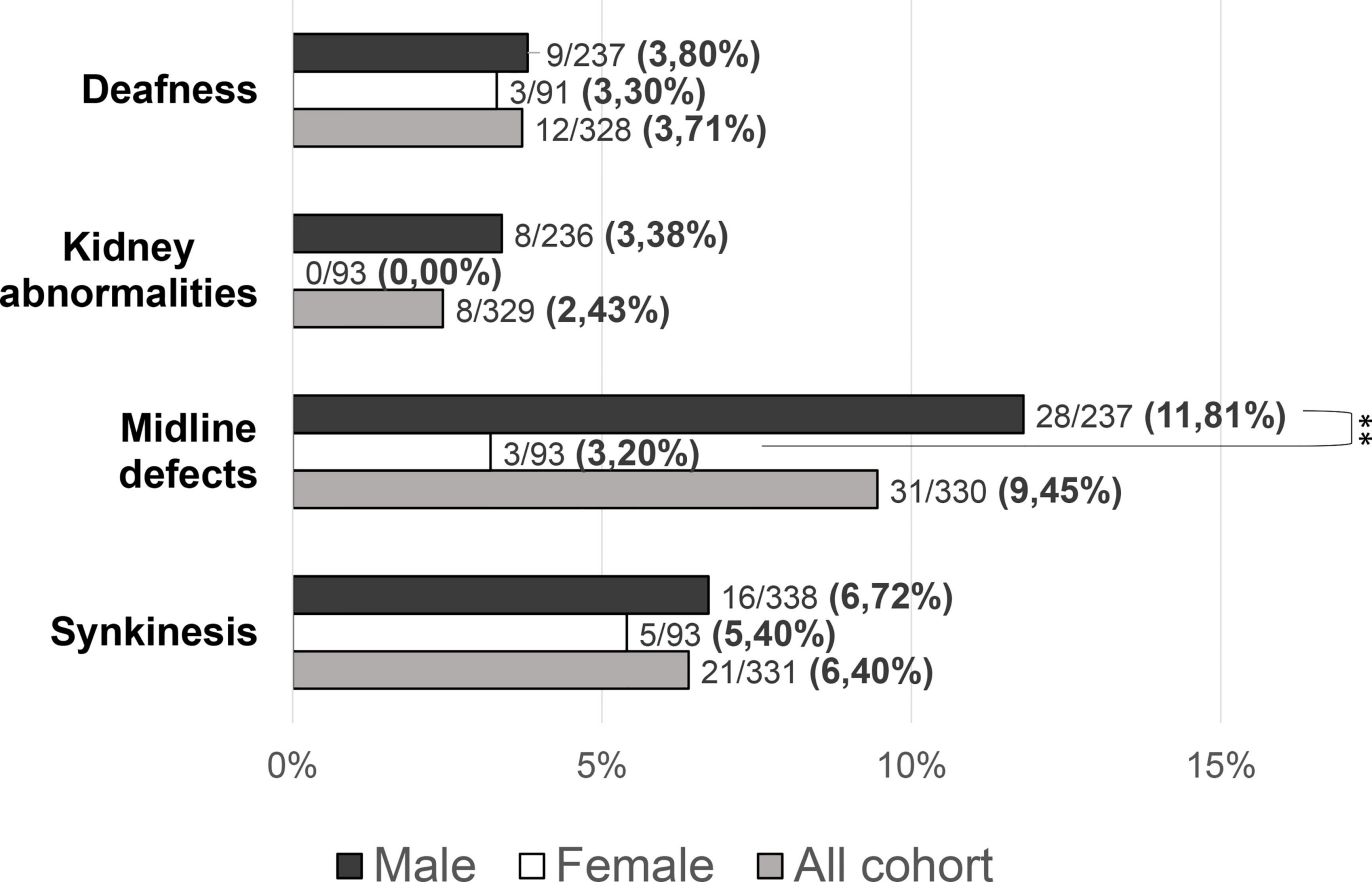
Prevalence of “non-reproductive” defects in the study cohort. Comparison between male and female patients using Fisher’s exact test: **P = <0.01.

Patients with at least one “red flag”, as in reproductive or non-reproductive phenotypic features associated with CHH (without considering the presence of anosmia), were 146/331 (48.33%). Such features were present in 136/238 (57.14%) male patients compared to only 10/93 (10.75%) females (*P =* <0.001). This prevalence was significantly higher in patients with KS (73/144, 50.69%) than in those with nCHH (73/187, 39.04%) (*P =* 0.022), and in those harboring rare genetic variants (64/117, 54.70%) compared to patients with wild-type gene sequences (82/214, 38.32%) (*P =* 0.005). When considering only the non-reproductive phenotypic features, excluding cryptorchidism and microphallus (signs of absent mini-puberty that occur only in males), the prevalence of “red flags” was still significantly higher (*P =* 0.01) in males (53/228, 23.24%) than in females (10/93, 10.75%) (overall 63/331; 19.0%). Also in this case, the prevalence of “red flags” was higher in patients with KS (43/144, 22.1%) than in those with nCHH (20/187, 10.7%) (*P =* <0.001) but only a trend toward a statistically significant difference was found in patients with an enrichment in rare variants compared to those with wild-type gene sequences (*P =* 0.057). The prevalence of “red flag” features associated with CHH, split into male and female population, is reported in **Figure 4**. The prevalence of microphallus in males was 52/203 (25.6%) and the prevalence of history of cryptorchidism was 99/231 (42.9%), with no significant difference according to diagnosis or presence of rare variants, even though there was a trend toward a greater enrichment in RVs (*P =* 0.05; *P =* 0.095 respectively). The prevalence of single non-reproductive characteristics was also evaluated. Deafness was present in 12/328 (3.71%) subjects, with no significant differences according to sex. Kidney abnormalities were present in 8/328 (2.43%) subjects and were found only in males, with a significantly higher prevalence in patients with KS than in those with nCHH (*P =* 0.023); among patients with kidney abnormalities, those harboring RVs were 5/8 and they all involved *ANOS1.* Midline defects were present in 31/336 (9.45%) subjects, with a significantly higher prevalence in male patients (*P =* 0.010) and KS patients (*P =* 0.007); among patients with midline defects and harboring RVs, 3/12 RVs were in *ANOS1* and 5/12 were in *FGFR1* (fibroblast growth factor receptor 1). The higher male prevalence of midline defects is maintained even after excluding patients with RVs within *ANOS1*. Bimanual synkinesis were present in 21/328 (6.40%) subjects, with no significant differences according to sex, but with a significantly higher prevalence in patients with KS than in those with nCHH (*P =* 0.007). The prevalence of each of the non-reproductive features associated with CHH, split into male and female populations, is reported in **Figure 5**.

**Figure 6:**
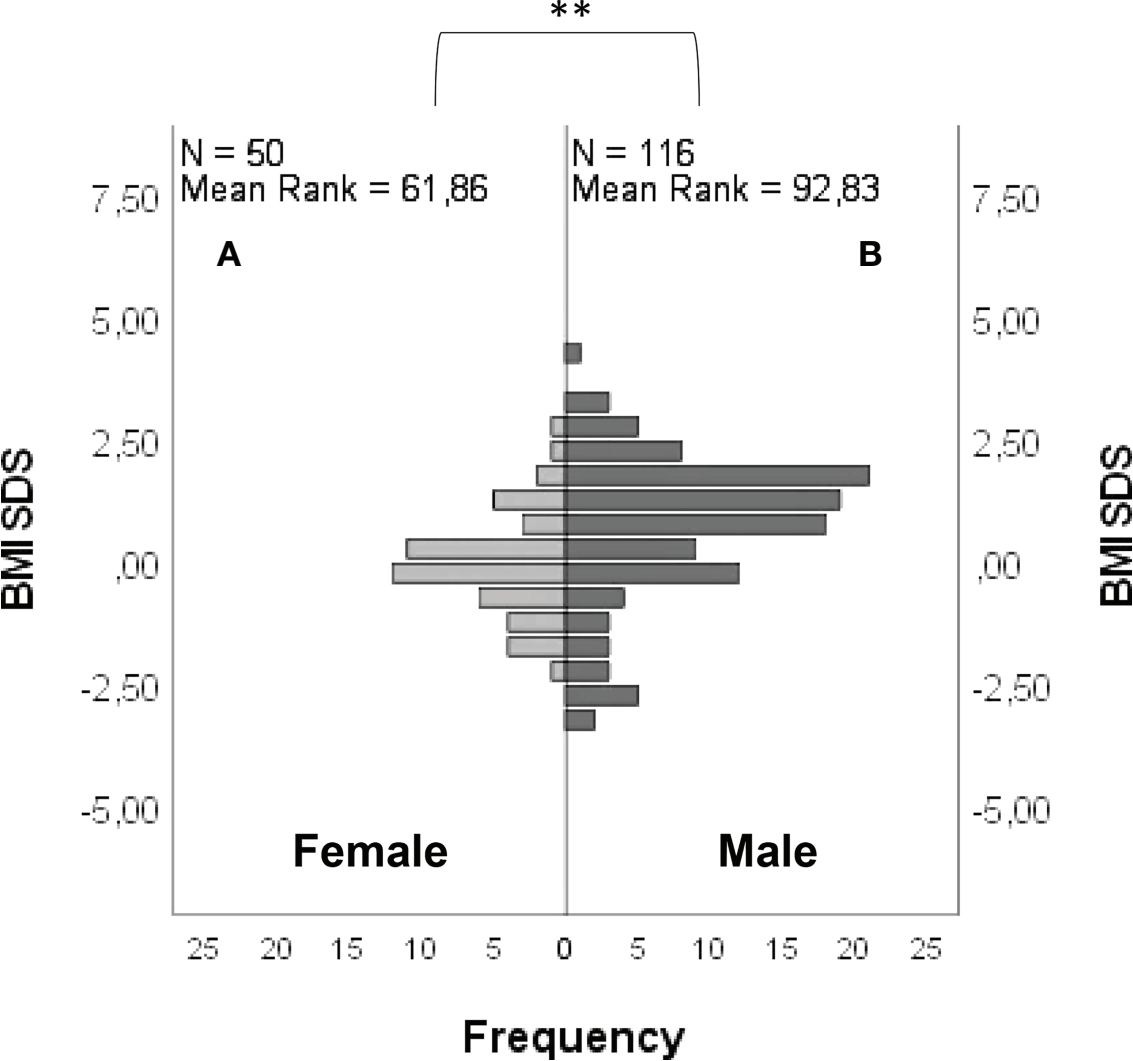
Frequency distribution of BMI SDS in female **(A)** and male **(B)** patients. Comparison between male and female cohorts using *t*-test: **P = <0.01.

BMI SDS was -0.04 ± 1.09 in females and 0.69 ± 1.51 in males (**Figure 6**), with a statistically significant difference between groups (*P =* <0.001). The age at diagnosis was 17.13 ± 2.82 for females and 16.75 ± 3.22 for males, with no statistically significant differences between groups (*P =* 0.06). A lower age at diagnosis was found in subjects with “red flags” (*P =* 0.01). No significant differences were found between BMI SDS and age at diagnosis in subjects carrying rare variants in candidate genes compared with those who did not.

### Female genotype and phenotype

**Figure 7:**
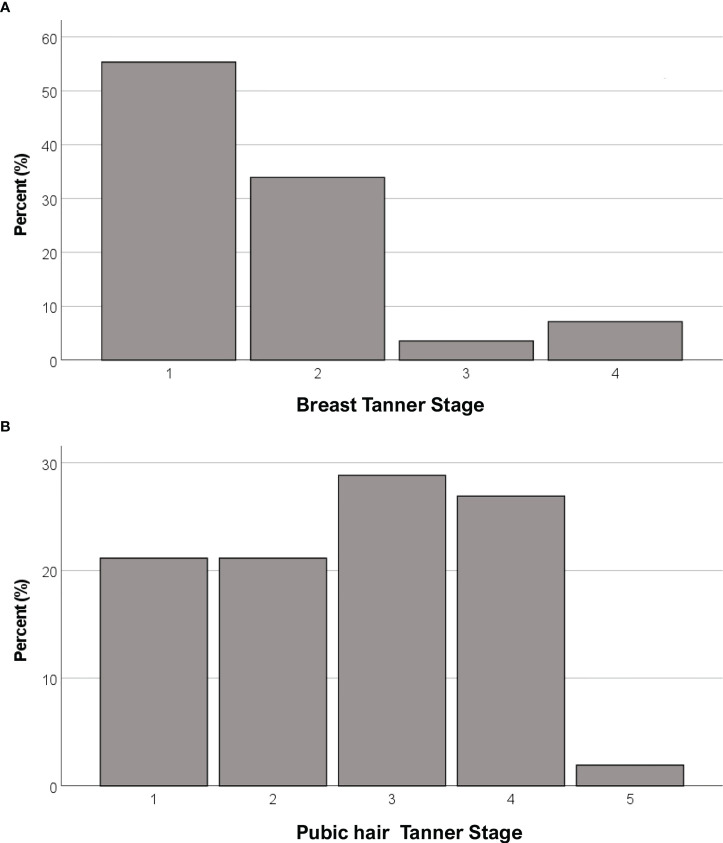
Distribution of female CHH patients for Breast Tanner Stages **(A)** and Pubic hair Tanner Stage **(B)**.

**Figure 8:**
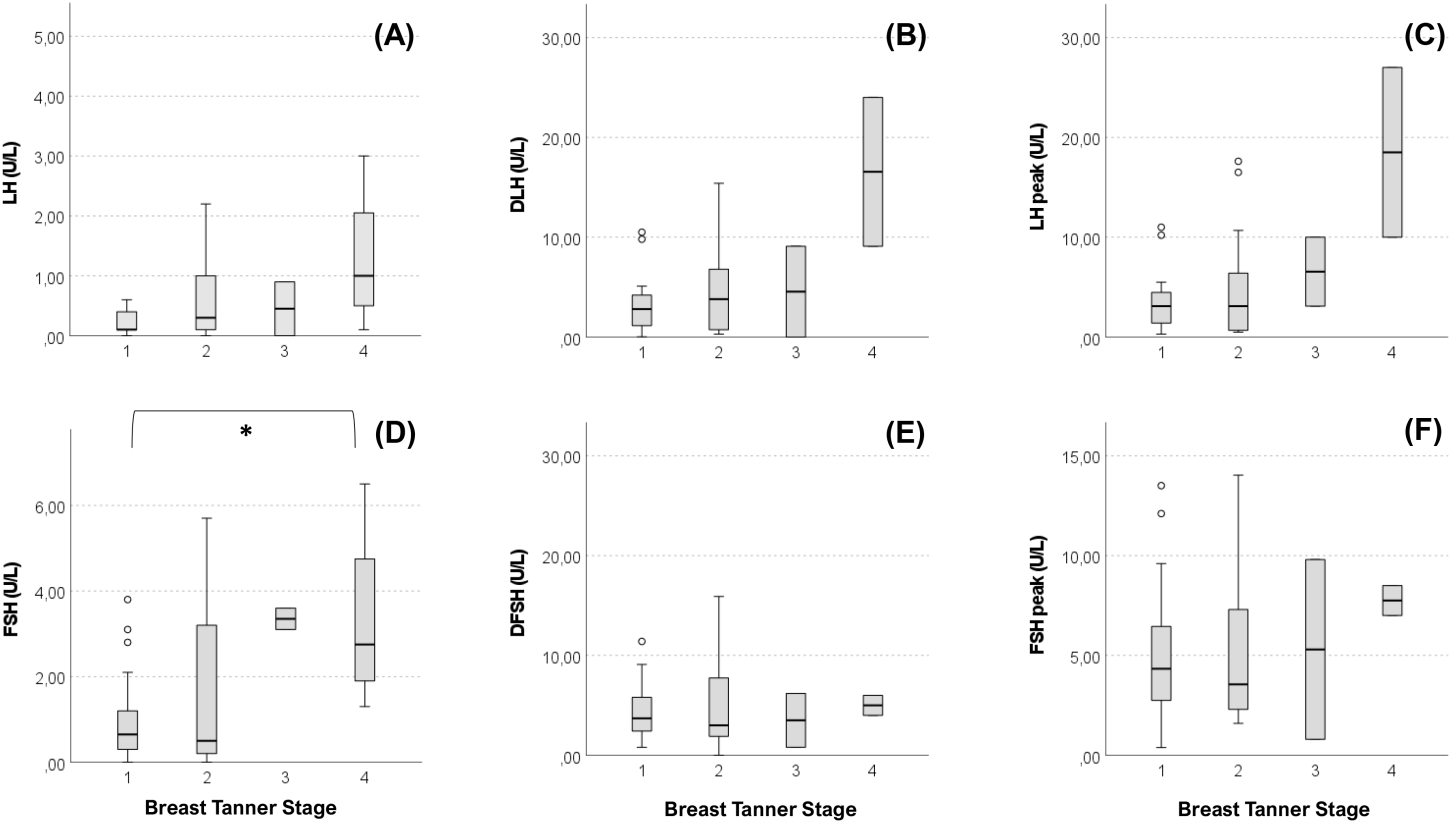
Basal and stimulated values of gonadotropins according Breast Tanner Stage. Value of basal LH **(A)**, difference between basal LH and LH peak (DLH) after stimulation test **(B)**, LH peak after stimulation test **(C)**, basal FSH **(D)**, difference between basal FSH and FSH peak (DFSH) after stimulation test **(E)**, FSH peak after stimulation test **(F)** compared according to Breast Tanner Stage. Pairwise comparison: *P = <0.05.

Anthropometric and hormonal parameters of the female cohort are shown in **Table 1**. The distribution of BMI we observed was comparable with that of the general population (21). No statistically significant differences were found in either hormonal or anthropometric parameters between KS and nCHH patients. Likewise, these parameters were no different between patients either harboring rare variants or not. Finally, patients with clinical “red flags” (deafness, kidney abnormalities, midline defects and bimanual synkinesis) showed only a shorter uterine length (*P =* 0.018) compared to the others. Distribution for Tanner stages is shown in **Figure 7**. Breast Tanner Stage and Pubic hair Tanner Stage are significantly correlated (*P =* <0.001). No statistically significant difference was found in Tanner stages at diagnosis according to either the presence of red flags at diagnosis or the enrichment in RVs. Breast Tanner Stage had a significant positive correlation with LH serum level (*P =* 0.04), ΔLH (*P =* 0.01), LH peak at LHRH stimulation test (*P =* <0.0001) and FSH serum level (*P =* <0.001) to linear regressions. However, when we compared hormonal values according to Breast Tanner Stage using non-parametric group comparison, the differences in both basal and stimulated LH did not result to be statistically significant, but we still found a significant difference in basal FSH values (*P =* 0.036) and 17βE_2_ (*P =* 0.041). The hormonal values according to Breast Tanner Stage are shown in **Figure 8**.

**Table 1:** Anthropometric and hormonal parameters of female patients.

	n	Range (min; max)	Mean ± SD
Anthropometric parameters			
Age (year)	88	10.00; 32.00	17.13 ± 2.82
Height (cm)	52	130.20; 177.00	158.81 ± 9.11
Height SDS	50	-2.88; 2.13	-0.53 ± 1.06
Weight (kg)	53	29.30; 88.50	54.35 ± 12.51
BMI (kg/m^2^)	75	15.50; 36.40	22.46 ± 4.33
BMI SDS	50	-2.15; 2.79	-0.04 ± 1.09
Biochemical parameters			
LH (U/L)	82	0.10; 6.50	0.57 ± 1.00
ΔLH (U/L)	57	0.00; 25.20	4.12 ± 5.17
LH peak (U/L)	57	0.00; 27.00	4.28 ± 5.07
FSH (U/L)	83	0.10; 7.20	1.50 ± 1.70
ΔFSH (U/L)	58	0.10; 29.00	4.76 ± 4.60
FSH peak (U/L)	55	0.39; 14.03	5.31 ± 3.62
17βE_2_ (pg/mL)	79	4.90; 87.00	11.78 ± 14.50
Imaging			
Uterine length (mm)	41	10.00; 64.00	35.67 ± 10.83

SDS, Standard Deviation Score; BMI, body mass index; LH, Luteinizing Hormone; ΔLH delta between basal LH and LH peak on stimulation testing. FSH, Follicle-Stimulating Hormone. ΔFSH delta between basal FSH and FSH peak on stimulation testing. 17βE_2_ Estradiol.

## Discussion

In our cohort of patients with CHH we found a female to male ratio of 1:2.6. This is broadly in line with the most recent studies (11–14) and family cases sub-analyses (10), but at the same time it is different from what was described in older studies (7–10).

By using a custom NGS panel of 28 candidate genes, the prevalence of RVs as well as the rate of oligogenicity are consistent with the current literature. We found that 36.09% of patients harbored potentially pathogenic rare genetic variants, 35,11% among females and 36.48% among males respectively: the prevalence of RVs between the two sexes was not significantly different, with no evidence of a different contribution in the genetic background on the development of the disease. In furtherance, our cohort analysis managed to reveal a difference in the genetic assortment of RVs by sex, whereby females with CHH exhibit oligogenic or biallelic variants more frequently than their male counterparts (even excluding *ANOS1*). On the other hand, there were no significant differences in the enrichment in individual variants (except for those within the X-linked gene *ANOS1*, limited to males only) although this result could also be due to the limited numerosity. The interpretation of these results is not simple, but they might hint for a stronger offsetting at the GnRH neurons level in females, such that more than one variant, or more destructive variants, are needed in order for the phenotype to occur. Still, it is recognized that the neuroendocrine reproductive axis differs between sexes in several remarkable ways, including its earlier activation in females at the time of pubertal maturation, the presence of neural circuitry that generates preovulatory hormone surges in females but not males, and the display of various sexually dimorphic reproductive behaviors (22). In particular, sex differences in the organization of kisspeptin neurons were described in rodents as the consequence of early perinatal actions of sex hormones (22–26); consistently, kisspeptin-immunoreactive neurons in humans were visualized within the preoptic region (which is involved in the positive feedback of sex steroids leading to the pre-ovulatory LH surge) only in females (24), who also seem to have a greater number of kisspeptin neurons within the arcuate nucleus (in which these cells drive the pulsatile GnRH secretion) compared to men (22, 25). However, many of the aspects of sex dimorphism in GnRH network are yet to be elucidated and may contribute to the variable expression of this disease. Another possible explanation for this sex difference is that female subjects who come to specialistic medical attention could be those with a more severe phenotype, due to a greater difficulty in recognising the CHH diagnosis, thus more likely harboring a more disrupting genetic assortment.

Moreover, several disparities between the two sexes were detected in the clinical presentation. First, we found an important difference in the prevalence of the so-called “red flags”. In particular, as previously reported in literature (9, 12), kidney malformations were found only in male patients, and they were associated exclusively with RVs in *ANOS1*. In addition, midline defects in our cohort were significantly more common among males rather than females: such defects are typical of patients harboring deleterious variants within *FGF8* and *FGFR1* (27), while we also noticed an enrichment in rare variants within *ANOS1* and *FGFR1* among these patients. However, the higher prevalence of midline defects is maintained even after excluding patients harboring *ANOS1* RVs from the analysis: hence, this difference does not seem to depend on a wider involvement of an X-linked gene among males. Thus, we propose the possibility that the aforementioned sexual dimorphism within the GnRH network and – consequently – in the expression of RVs in CHH-associated genes could explain these phenotypical differences.

When we compared the SDS BMI between our female and male CHH patients, the former resulted significantly lower. In particular, while the distribution of BMI (namely SDS BMI) in our female cohort resulted broadly in line for age and sex with the reference population, males tended toward a higher BMI (21). Indeed, it is well known that body weight influences pubertal onset and reproductive function. It is established that an excessive weight in females determines pubertal advancement, while an energy deficiency can cause a functional amenorrhea; on the other hand, pubertal onset in males could be influenced by the degree of weight gain, with an earlier maturation in overweight subjects and a delayed maturation in obese ones (28). In a population of obese males with adult-onset hypogonadism it has also been pointed out that a mild form of GnRH deficiency can be characterized by a genetic origin that frequently overlaps with that of severe CHH, and obesity could be only one of the acquired cofactors involved in the onset of hypogonadism among adult subjects that are naturally prone to develop a central failure of the gonadal axis (29). Our findings are in line with the idea that patients’ phenotype is the result of a complex interaction between genetic factors and metabolic/behavioral factors, with the latter having a variable influence according to sex.

Patients with CHH often have a delayed diagnosis, and such delay is particularly remarkable in females, considering the earlier physiological timing of their pubertal onset compared to boys. On the one hand, the wider diagnostic delay in girls could be explained in the light of the higher prevalence of “red flags” in males, which may facilitate the diagnostic process and therefore lead to an earlier recognition of this condition. On the other hand, this might be also justified by an insufficient awareness of this disease among gynecologists or general pediatricians that more frequently deal with primary or functional hypothalamic amenorrhea.

Regarding clinical presentation in female patients, in contrast to previous reports (30), no differences emerged between female patients with KS and nCHH. In addition, no relevant biochemical and anthropometric differences were found based on the presence of “red flags”, aside for a shorter uterine length associated with non-reproductive characteristics. More than 50% of our patients were completely pre-pubertal at the time of diagnosis, but in the other cases a variable degree of pubertal advancement was observed before its arrest. From a biochemical point of view this is mainly revealed by a more pronounced LH response to dynamic testing, as observed in cases of spontaneous pubertal onset, although basal LH values remain inappropriately reduced. As can be noted from **Figure 8**, while a predominant FSH response over LH is maintained in patients with Breast Tanner Stage 1, which is typical of a prepubertal condition, in patients with spontaneous pubertal onset and subsequent arrest this ratio is inverted, which is an occurrence compatible with a previous activation of the HPG axis. Basal FSH values, on the other hand, seem to correlate better with pubertal advancement. However, these observations corroborate the concept of CHH as a disease with a broad clinical scenario, suggesting a possible reappraisal of those clinical forms considered spurious in the past.

The robustness of these results is constrained mainly by the limited sample size, and from the potential bias that data come from a single Centre. However, in consideration of the rarity of this disease, ours is one of the largest series ever studied with such a wide panel of candidate genes: we therefore believe that this study may still have some relevance in the current state of knowledge. Moreover, it must be acknowledged that despite our efforts in providing an accurate pathogenicity classification of the identified rare variants according to the available guidelines, the attribution of an etiologic significance to each variant is very challenging, especially in the context of oligogenicity: in this case, in fact, even rare variants theoretically considered insufficient to cause the disease by themselves could instead contribute additively in affecting the clinical phenotype.

In conclusion, despite the analysis of this large CHH cohort does not clearly point out striking differences in genetic background according to sex, intriguingly it unveils a greater prevalence of oligogenicity in females. In addition, a greater prevalence of non-reproductive phenotypic characteristics and a higher BMI emerge in male patients compared to females. These findings identify some distinctions in the clinical presentation between the two sexes that could indicate a variable expression of genetic rare variants and a dimorphic modulation of the phenotype according to metabolic/behavioral factors, which will need to be substantiated and investigated by further studies. On the other hand, we can assume that many of the epidemiological differences observed between males and females might depend on the lack of specialist diagnostic attention in a proportion of girls with delayed puberty, which could constitute a *selection bias* in cohort analysis. It is likely that the refinement of diagnostic sensitivity in recent years might explain the decrease of such gap between sexes which was observed among the latest studies.

## Data availability statement

The datasets presented in this study can be found in online repositories. The names of the repository/repositories and accession number(s) can be found below: https://zenodo.org/record/6600520#.YpZ4Y1RBzb0, Zenodo database.

## Ethics statement

The studies involving human participants were reviewed and approved by Istituto Auxologico Italiano ethics committee. Written informed consent to participate in this study was provided by the participants’ legal guardian/next of kin.

## Author contributions

SF, BC, MB contributed to the conception, data analysis and first draft of the manuscript. MB, LP, BC, SF, GG, LG performed patient follow-up, clinical diagnosis, and treatment. MB, SF, DM, EM, MA collected clinical data. MB, LP, VV, FH were responsible for genetic analysis and its interpretation MB, LP, BC, VV contributed to the supervision and critical revision of the manuscript. All authors contributed to the article and approved the submitted version.

## Funding

Research funded by the Italian Ministry of Health, Young Investigators funds: (GR-2016- 02362389) IRCCS Istituto Auxologico Italiano (Ricerca Corrente funds: O5C202_2012), and Dept. of Medical Biotechnology and Tranlational Medicine - University of Milan (PSR2020_BONOMI_LINEA_C).

## Acknowledgments

SF is presently recipient of a “Type-B Research Fellowship” funded by the Department of Medical Biotechnology and Translational Medicine, University of Milan.

## Conflict of interest

The authors declare that the research was conducted in the absence of any commercial or financial relationships that could be construed as a potential conflict of interest.

## Publisher’s note

All claims expressed in this article are solely those of the authors and do not necessarily represent those of their affiliated organizations, or those of the publisher, the editors and the reviewers. Any product that may be evaluated in this article, or claim that may be made by its manufacturer, is not guaranteed or endorsed by the publisher.
