# Supplementary material for: Genetic and phenotypic differences between sexes in congenital hypogonadotropic hypogonadism (CHH): Large cohort analysis from a single tertiary centre
Source: Front Endocrinol (Lausanne). 2022 Dec 2;13:965074. doi: 10.3389/fendo.2022.965074 (PMC9755160; doi:10.3389/fendo.2022.965074)
Supplement: Supplementary file 1 [file Table_1.docx]

| ID | diagnosis | family history | gene | zygosity | inheritance | rsID | transcript | HGVS coding | HGVS protein | coding impact | ClinVar | ACMG classification | MAF | MAF (ref pop) | |
| --- | --- | --- | --- | --- | --- | --- | --- | --- | --- | --- | --- | --- | --- | --- | --- |
| 4 | nCHH | yes | *CHD7* | Het | AD | [rs1244745262](https://varsome.com/variant/hg38/rs1244745262?&annotation-mode=germline) | NM_017780.4 | c.5713A>G | N1905D (p.Asn1905Asp) | missense | nd | Likely benign | 0.00000657 | 0.0000147 |  |
| 4 | nCHH | yes | *SPRY4* | Het | AR | [rs568363732](https://varsome.com/variant/hg38/rs568363732?&annotation-mode=germline) | NM_030964.5 | c.313G>A | D105N (p.Asp105Asn) | missense | Likely benign | Likely benign | 0.000158 | 0.0000747 |  |
| 21 | nCHH | no | *CHD7* | Het | AD | nd | NM_017780.4 | c.4012G>A | G1338S (p.Gly1338Ser) | missense | nd | Likely pathogenic | nd | nd |  |
| 21 | nCHH | no | *PROKR2* | Het | AD/AR | [rs74315416](https://varsome.com/variant/hg38/rs74315416?&annotation-mode=germline) | NM_144773.4 | c.518T>G | L173R (p.Leu173Arg) | missense | Conflicting | Likely pathogenic | 0.00212 | 0.00347 |  |
| 24 | nCHH | no | *PROKR2* | Het | AD/AR | [rs74315416](https://varsome.com/variant/hg38/rs74315416?&annotation-mode=germline&zygosity=Heterozygous&sex=F&family-members-also-affected=False&family-segregation=Unknown) | NM_144773.4 | c.518T>G | L173R (p.Leu173Arg) | missense | Conflicting | Likely pathogenic | 0.00212 | 0.00347 |  |
| 24 | nCHH | no | *SEMA3A* | Het | AD | [rs147436181](https://varsome.com/variant/hg38/rs147436181?&annotation-mode=germline&zygosity=Heterozygous&sex=F&family-members-also-affected=False&family-segregation=Unknown) | NM_006080.3 | c.1303G>A | V435I (p.Val435Ile) | missense | Benign | Benign | 0.0125 | 0.0215 |  |
| 33 | nCHH | no* | *SEMA3E* | Het | AD | [rs773917768](https://varsome.com/variant/hg38/rs773917768?&annotation-mode=germline&zygosity=Heterozygous&sex=F&family-members-also-affected=False&family-segregation=Unknown) | NM_012431.3 | c.781G>A | A261T (p.Ala261Thr) | missense | Uncertain significance | Likely benign | 0.0000319 | 0.0000529 |  |
| 33 | nCHH | no* | *WDR11* | Het | AD | [rs74870997](https://varsome.com/variant/hg38/rs74870997?&annotation-mode=germline&diseases=nCHH&zygosity=Heterozygous&family-members-also-affected=False&family-segregation=Unknown) | NM_018117.12 | c.2962G>A | E988K (p.Glu988Lys) | missense | Likely benign | Benign | 0.00144 | 0.00301 |  |
| 34 | nCHH | yes* | *KISS1R* | Hom | AR | [rs104894702](https://varsome.com/variant/hg38/rs104894702?&annotation-mode=germline&zygosity=Homozygous&family-members-also-affected=True) | NM_032551.5 | c.1195T>A | *399Rext* (p.Ter399Argext*) | stopLoss | Likely pathogenetic | Pathogenic | 0.0000681 | 0.000112 |  |
| 35 | nCHH | yes | *KISS1R* | Hom | AR | nd | NM_032551.5 | c.555del | P187Rfs*132 (p.Pro187ArgfsTer132) | frameshift | nd | Likely pathogenic | nd | nd |  |
| 35 | nCHH | yes | *SOX10* | Het | AD | [rs766087219](https://varsome.com/variant/hg38/rs766087219?&annotation-mode=germline&zygosity=Heterozygous&family-members-also-affected=True&family-segregation=Unknown) | NM_006941.4 | c.967G>A | A323T (p.Ala323Thr) | missense | nd | Uncertain significance | 0.00000404 | 0.00000884 |  |
| 37 | nCHH | yes* | *TACR3* | Hom | AR | [rs144292455](https://varsome.com/variant/hg38/rs144292455?&annotation-mode=germline&zygosity=Homozygous&family-members-also-affected=True&family-segregation=Unknown) | NM_001059.3 | c.824G>A | W275* (p.Trp275Ter) | nonsense | Pathogenic | Pathogenic | 0.000307 | 0.000626 |  |
| 56 | nCHH | no* | *CHD7* | Het | AD | [rs184814820](https://varsome.com/variant/hg38/rs184814820?&annotation-mode=germline&zygosity=Heterozygous&inheritance=Confirmed%20De%20Novo&family-members-also-affected=False) | NM_017780.4 | c.8950C>T | L2984F (p.Leu2984Phe) | missense | Likely benign | Benign | 0.00393 | 0.00657 |  |
| 57 | nCHH | no | *PROKR2* | Het | AD/AR | [rs141090506](https://varsome.com/variant/hg38/rs141090506?&annotation-mode=germline&zygosity=Heterozygous&family-members-also-affected=False&family-segregation=Unknown) | NM_144773.4 | c.253C>T | R85C (p.Arg85Cys) | missense | Conflicting | Likely Benign | 0.000592 | 0.000369 |  |
| 64 | nCHH | yes* | *FGFR1* | Het | AD | [rs397515446](https://varsome.com/variant/hg38/rs397515446?&annotation-mode=germline&zygosity=Heterozygous&family-members-also-affected=True) | NM_023110.3 | c.2008G>A | E670K (p.Glu670Lys) | missense | Risk factor | Pathogenic | nd | nd |  |
| 97 | nCHH | no* | *ANOS1* | Het | XLinked | [rs141239804](https://varsome.com/variant/hg38/rs141239804?&annotation-mode=germline&zygosity=Heterozygous&sex=F&family-members-also-affected=False) | NM_000216.4 | c.890G>A | R297Q (p.Arg297Gln) | missense | Benign | Benign | 0.000198 | nd |  |
| 98 | nCHH | no | *CHD7* | Het | AD | [rs767259131](https://varsome.com/variant/hg38/rs767259131?&annotation-mode=germline&zygosity=Heterozygous&family-members-also-affected=False) | NM_017780.4 | c.3299G>A | R1100H (p.Arg1100His) | missense | Uncertain significance | Likely benign | 0.0000562 | 0.000062 |  |
| 98 | nCHH | no | *KISS1R* | Het | AR | [rs1440702117](https://varsome.com/variant/hg38/rs1440702117?&annotation-mode=germline&zygosity=Heterozygous&family-members-also-affected=False) | NM_032551.5 | c.515C>T | A172V (p.Ala172Val) | missense | nd | Likely benign | 0.00000667 | 0.0000149 |  |
| 106 | nCHH | no | *HESX1* | Het | AD/AR | [rs781301558](https://varsome.com/variant/hg38/rs781301558?&annotation-mode=germline&zygosity=Heterozygous&family-members-also-affected=False) | NM_003865.3 | c.40A>G | N14D (p.Asn14Asp) | missense | nd | Likely benign | 0.00000398 | 0.00000879 |  |
| 116 | nCHH | yes | *CHD7* | Het | AD | nd | NM_017780.4 | c.824_825delTCinsGA | F275* (p.Phe275Ter) | nonsense | nd | Likely pathogenic | nd | nd |  |
| 121 | nCHH | no | *HS6ST1* | Het | AD | [rs200268730](https://varsome.com/variant/hg38/rs200268730?&annotation-mode=germline&zygosity=Heterozygous&family-members-also-affected=False) | NM_004807.3 | c.652C>T | P218S (p.Pro218Ser) | missense | Conflicting | Benign | 0.00183 | 0.00313 |  |
| 121 | nCHH | no | *SEMA3A* | Het | AD | [rs148900275](https://varsome.com/variant/hg38/rs148900275?&annotation-mode=germline&zygosity=Heterozygous&family-members-also-affected=False) | NM_006080.3 | c.1546C>T | R516W (p.Arg516Trp) | missense | nd | Likely benign | 0.0000919 | 0.0000973 |  |
| 125 | nCHH | no | *IL17RD* | Het | AR | [rs948312065](https://varsome.com/variant/hg38/rs948312065?&annotation-mode=germline&zygosity=Heterozygous&family-members-also-affected=False) | NM_017563.5 | c.68T>G | L23R (p.Leu23Arg) | missense | nd | Likely benign | 0.000049 | 0.000116 |  |
| 134 | nCHH | no | *FEZF1* | Het | AD/AR | [rs370467502](https://varsome.com/variant/hg38/rs370467502?&annotation-mode=germline&zygosity=Heterozygous&family-members-also-affected=False) | NM_001024613.4 | c.553T>C | F185L (p.Phe185Leu) | missense | Likely benign | Likely benign | 0.000567 | 0.000194 |  |
| 138 | nCHH | yes | *GNRHR* | CH (trans) | AR | nd | NM_000406.3 | c.742G>C | E248Q (p.Glu248Gln) | missense | nd | Likely pathogenic | nd | nd |  |
| 138 | nCHH | yes | *GNRHR* | CH (trans) | AR | nd | NM_000406.3 | c.987A>G | *329Wext*22 (p.Ter329Trpext*22) | stopLoss | nd | Uncertain significance | nd | nd |  |
| 142 | nCHH | yes | *FGFR1* | Het | AD | rs1563516081 | NM_023110.3 | c.156G>C | Q52H (p.Gln52His) | missense | nd | Uncertain significance | 0.000004 | 0.00000885 |  |
| 142 | nCHH | yes | *SEMA7A* | Het | AD | [rs1045470923](https://varsome.com/variant/hg38/rs1045470923?&annotation-mode=germline&zygosity=Heterozygous&family-members-also-affected=True) | NM_003612.5 | c.115G>A | A39T (p.Ala39Thr) | missense | nd | Likely benign | 0.0000197 | 0.0000441 |  |
| 144 | nCHH | no | *CHD7* | Het | AD | [rs759918327](https://varsome.com/variant/hg38/rs759918327?&annotation-mode=germline&zygosity=Heterozygous&family-members-also-affected=False) | NM_017780.4 | c.5512A>G | M1838V (p.Met1838Val) | missense | nd | Uncertain significance | 0.00000406 | nd |  |
| 144 | nCHH | no | *FGFR1* | Het | AD | nd | NM_023110.3 | c.1829G>T | G610V (p.Gly610Val) | missense | nd | Likely pathogenic | nd | nd |  |
| 144 | nCHH | no | *IL17RD* | Het | AR | [rs61740622](https://varsome.com/variant/hg38/rs61740622?&annotation-mode=germline&zygosity=Heterozygous&family-members-also-affected=False) | NM_017563.5 | c.1924G>C | A642P (p.Ala642Pro) | missense | Likely benign | Benign | 0.003 | 0.0359 |  |
| 151 | nCHH | yes* | *CHD7* | Het | AD | nd | NM_017780.4 | c.2908del | E970Nfs*7 (p.Glu970AsnfsTer7) | frameshift | nd | Likely pathogenic | nd | nd |  |
| 157 | nCHH | yes | *GNRH2* | Het | AR | [rs544846287](https://varsome.com/variant/hg38/rs544846287?&annotation-mode=germline&zygosity=Heterozygous&family-members-also-affected=True) | NM_021081.6 | c.326A>C | E109A (p.Glu109Ala) | missense | nd | Likely benign | 0.000101 | 0.0000466 |  |
| 161 | nCHH | yes* | *WDR11* | Het | AD | [rs144531702](https://varsome.com/variant/hg38/rs144531702?&annotation-mode=germline&zygosity=Heterozygous&family-members-also-affected=True) | NM_018117.12 | c.2932A>C | K978Q (p.Lys978Gln) | missense | Not provided | Benign | 0.000184 | 0.000345 |  |
| 163 | nCHH | no | *HESX1* | Het | AD/AR | [rs761470587](https://varsome.com/variant/hg38/rs761470587?&annotation-mode=germline&zygosity=Heterozygous&family-members-also-affected=False) | NM_003865.3 | c.124C>T | H42Y (p.His42Tyr) | missense | Uncertain significance | Likely pathogenic | 0.0000597 | 0.0000439 |  |
| 175 | nCHH | no* | *TACR3* | CH (trans) | AD/AR | [rs144292455](https://varsome.com/variant/hg38/rs144292455?&annotation-mode=germline&zygosity=Homozygous&sex=F&family-members-also-affected=False) | NM_001059.3 | c.824G>A | W275* (p.Trp275Ter) | nonsense | Pathogenic | Pathogenic | 0.000307 | 0.000626 |  |
| 175 | nCHH | no* | *TACR3* | CH (trans) | AD/AR | nd | NM_001059.3 | c.769T>C | C257R (p.Cys257Arg) | missense | nd | Uncertain significance | nd | nd |  |
| 183 | KS | no | *CHD7* | Het | AD | rs71640285 | NM_017780.4 | c.1397C>T | S466L (p.Ser466Leu) | missense | Conflicting | Benign | 0.00103 | 0.00191 |  |
| 191 | KS | yes | *SEMA3A* | Het | AD | [rs1441276545](https://varsome.com/variant/hg38/rs1441276545?&annotation-mode=germline&zygosity=Heterozygous&family-members-also-affected=False) | NM_006080.3 | c.1354G>A | G452R (p.Gly452Arg) | missense | nd | Uncertain significance | 0.00000658 | nd |  |
| 192 | KS | no* | *ANOS1* | Het | XLinked | [rs894572813](https://varsome.com/variant/hg38/rs894572813?&annotation-mode=germline&zygosity=Heterozygous&sex=M&inheritance=Mother&family-members-also-affected=False) | NM_000216.4 | c.740G>A | R247Q (p.Arg247Gln) | missense | nd | Likely benign | nd | nd |  |
| 195 | KS | no* | *ANOS1* | Het | XLinked | [rs755516273](https://varsome.com/variant/hg38/rs755516273?&annotation-mode=germline&diseases=Kallmann%20Syndrome%2CAnosmia&zygosity=Heterozygous&sex=F&inheritance=Mother&family-members-also-affected=False) | NM_000216.4 | c.397G>A | D133N (p.Asp133Asn) | missense | nd | Likely benign | 0.0000164 | 0.0000368 |  |
| 202 | KS | yes* | *CHD7* | Het | AD | [rs758402387](https://varsome.com/variant/hg38/rs758402387?&annotation-mode=germline&zygosity=Heterozygous&family-members-also-affected=True) | NM_017780.4 | c.470G>A | R157Q (p.Arg157Gln) | missense | nd | Uncertain significance | 0.00000814 | nd |  |
| 204 | KS | no* | *FGFR1* | Het | AD | nd | NM_023110.3 | c.1052A>G | H351R (p.His351Arg) | missense | nd | Likely pathogenic | nd | nd |  |
| 215 | KS | no | *FGFR1* | Het | AD | nd | NM_023110.3 | c.748C>T | R250W (p.Arg250Trp) | missense | Likely pathogenetic | Pathogenic | nd | nd |  |
| 215 | KS | no | *PROKR2* | Het | AD/AR | [rs774093318](https://varsome.com/variant/hg38/rs774093318?&annotation-mode=germline&diseases=Anosmia%2CHypogonadotropic%20Hypogonadism%2CKallmann%20Syndrome&zygosity=Heterozygous&family-members-also-affected=False) | NM_144773.4 | c.238C>T | R80C (p.Arg80Cys) | missense | nd | Uncertain significance | 0.00000795 | 0.00000879 |  |
| 218 | KS | no | *SEMA3A* | Het | AD | nd | NM_006080.3 | c.2009_2010del | T670Rfs*9 (p.Thr670ArgfsTer9) | frameshift | nd | Likely pathogenic | nd | nd |  |
| 228 | KS | no | *CHD7* | Het | AD | [rs763438086](https://varsome.com/variant/hg19/rs763438086?&annotation-mode=germline&diseases=Anosmia%2CHypogonadotropic%20Hypogonadism%2CKallmann%20Syndrome&zygosity=Heterozygous&family-members-also-affected=False) | NM_017780.4 | c.2024A>G | K675R (p.Lys675Arg) | missense | nd | Uncertain significance | nd | nd |  |
| 237 | KS | yes | *WDR11* | Het | AD | [rs144531702](https://varsome.com/variant/hg19/rs144531702?&annotation-mode=germline&diseases=Anosmia%2CHypogonadotropic%20Hypogonadism%2CKallmann%20Syndrome&zygosity=Heterozygous&family-members-also-affected=True) | NM_018117.12 | c.2932A>C | K978Q (p.Lys978Gln) | missense | Not provided | Benign | 0.000184 | 0.000345 |  |
| 245 | KS | no* | *SOX10* | Het | AD | nd | NM_006941.4 | c.355C>T | R119C (p.Arg119Cys) | missense | nd | Likely pathogenic | nd | nd |  |
| 246 | KS | no | *FGFR1* | Het | AD | nd | NM_023110.3 | c.1981C>T | R661* (p.Arg661Ter) | nonsense | Pathogenic | Pathogenic | nd | nd |  |
| 251 | KS | yes | *FGFR1* | Het | AD | nd | NM_023110.3 | c.820G>T | E274* (p.Glu274Ter) | nonsense | nd | Likely pathogenic | nd | nd |  |
| 267 | KS | no | *CHD7* | Het | AD | rs71640285 | NM_017780.4 | c.1397C>T | S466L (p.Ser466Leu) | missense | Conflicting | Benign | 0.00103 | 0.00191 |  |
| 267 | KS | no | *PROKR2* | Het | AD/AR | [rs74315416](https://varsome.com/variant/hg38/rs74315416?&annotation-mode=germline) | NM_144773.4 | c.518T>G | L173R (p.Leu173Arg) | missense | Conflicting | Likely pathogenic | 0.00212 | 0.00347 |  |
| 267 | KS | no | *WDR11* | Het | AD | rs776634452 | NM_018117.12 | c.3319G>A | D1107N (p.Asp1107Asn) | missense | nd | Uncertain significance | 0.0000159 | 0.0000264 |  |
| 286 | KS | yes* | *FGFR1* | Het | AD | [rs121909628](https://varsome.com/variant/hg38/rs121909628?&annotation-mode=germline&zygosity=Heterozygous&sex=F&inheritance=Confirmed%20De%20Novo&family-members-also-affected=True) | NM_023110.3 | c.1864C>T | R622* (p.Arg622Ter) | nonsense | Pathogenic | Pathogenic | nd | nd |  |
| 288 | KS | yes* | *FGFR1* | Het | AD | nd | NM_023110.3 | c.760C>T | R254W (p.Arg254Trp) | missense | nd | Likely pathogenic | nd | nd |  |
| 288 | KS | yes* | *FLRT3* | Het | AD | [rs774538592](https://varsome.com/variant/hg38/rs774538592?&annotation-mode=germline&zygosity=Heterozygous&family-members-also-affected=True&family-segregation=False) | NM_198391.3 | c.1865T>C | L622P (p.Leu622Pro) | missense | nd | uncertain significance | 0.0000159 | 0.0000176 |  |
| 299 | KS | no* | *GNRHR* | CH (trans) | AR | [rs104893836](https://varsome.com/variant/hg38/rs104893836?&annotation-mode=germline&zygosity=Homozygous&sex=F&inheritance=Mother&family-members-also-affected=False) | NM_000406.3 | c.317A>G | Q106R (p.Gln106Arg) | missense | Pathogenic | Pathogenic | 0.00284 | 0.00418 |  |
| 299 | KS | no* | *GNRHR* | CH (trans) | AR | [rs104893842](https://varsome.com/variant/hg38/rs104893842?&annotation-mode=germline&zygosity=Homozygous&sex=F&inheritance=Both&family-members-also-affected=False) | NM_000406.3 | c.416G>A | R139H (p.Arg139His) | missense | Pathogenic | Pathogenic | 0.000144 | 0.000168 |  |
| 301 | KS | yes | *FGFR1* | Het | AD | [rs886037634](https://varsome.com/variant/hg38/rs886037634?&annotation-mode=germline&zygosity=Heterozygous&sex=F&family-members-also-affected=True) | NM_023110.3 | c.1042G>A | G348R (p.Gly348Arg) | missense | Pathogenic | Pathogenic | nd | nd |  |
| 301 | KS | yes | *PROKR2* | Het | AD/AR | [rs74315416](https://varsome.com/variant/hg38/rs74315416?&annotation-mode=germline) | NM_144773.4 | c.518T>G | L173R (p.Leu173Arg) | missense | Conflicting | Likely pathogenic | 0.00212 | 0.00347 |  |
| 305 | KS | yes* | *FGFR1* | Het | AD | nd | NM_023110.3 | c.1871T>A | L624Q (p.Leu624Gln) | missense | nd | Likely pathogenic | nd | nd |  |
| 312 | KS | no* | *GNRH1* | Het | AR | nd | NM_000825.3 | c.103C>T | R35C (p.Arg35Cys) | missense | nd | Pathogenic | nd | nd |  |
| 315 | nCHH | no | *FGFR1* | Het | AD | [rs1329256283](https://varsome.com/variant/hg38/rs1329256283?&annotation-mode=germline&zygosity=Heterozygous&family-members-also-affected=False) | NM_023110.3 | c.2200C>T | R734W (p.Arg734Trp) | missense | Uncertain significance | Uncertain significance | 0.00000799 | 0.00000882 |  |
| 315 | nCHH | no | *GNRHR* | Hom | AR | nd | NM_000406.3 | c.378T>A | Y126* (p.Tyr126Ter) | nonsense | nd | Likely pathogenic | nd | nd |  |
| 318 | nCHH | yes | *PROKR2* | Het | AD/AR | [rs74315416](https://varsome.com/variant/hg38/rs74315416?&annotation-mode=germline) | NM_144773.4 | c.518T>G | L173R (p.Leu173Arg) | missense | Conflicting | Likely pathogenic | 0.00212 | 0.00347 |  |
| 320 | nCHH | nd | *FEZF1* | Het | AD/AR | rs765509411 | NM_001024613.4 | c.1372C>T | P458S (p.Pro458Ser) | missense | nd | Likely benign | 0.00000423 | 0.00000936 |  |
| 320 | nCHH | nd | *FGFR1* | Het | AD | nd | NM_023110.3 | c.165_171dup | R58Sfs*55 (p.Arg58SerfsTer55) | frameshift | nd | Likely pathogenic | nd | nd |  |
| 320 | nCHH | nd | *TAC3* | Het | AD/AR | nd | NM_013251.4 | c.52A>C | S18R (p.Ser18Arg) | missense | nd | Likely benign | nd | nd |  |
| 333 | nCHH | no | *FEZF1* | Het | AD/AR | rs1454327695 | NM_001024613.4 | c.722T>A | I241N (p.Ile241Asn) | missense | nd | Uncertain significance | 0.00000398 | 0.00000879 |  |
| 333 | nCHH | no | *GNRHR* | CH (cis) | AR | [rs200026876](https://varsome.com/variant/hg38/rs200026876?&annotation-mode=germline&zygosity=Heterozygous&family-members-also-affected=False) | NM_000406.3 | c.718C>T | R240W (p.Arg240Trp) | missense | nd | Uncertain significance | 0.00000398 | nd |  |
| 333 | nCHH | no | *GNRHR* | CH (cis) | AR | [rs104893836](https://varsome.com/variant/hg38/rs104893836?&annotation-mode=germline&zygosity=Heterozygous&family-members-also-affected=False) | NM_000406.3 | c.317A>G | Q106R (p.Gln106Arg) | missense | Pathogenic | Pathogenic | 0.00284 | 0.00199 |  |
| 334 | nCHH | no | *CHD7* | Het | AD | [rs1386245550](https://varsome.com/variant/hg38/rs1386245550?&annotation-mode=germline&zygosity=Heterozygous&sex=F&family-members-also-affected=False) | NM_017780.4 | c.5932G>A | V1978I (p.Val1978Ile) | missense | nd | Uncertain significance | 0.00000657 | 0.0000147 |  |
| 342 | KS | no* | *FGFR1* | Het | AD | nd | NM_023110.3 | c.835G>A | V279M (p.Val279Met) | missense | nd | Likely benign | nd | nd |  |

| ID | diagnosis | family history | gene | zygosity | inheritance | rsID | transcript | HGVS coding | HGVS protein | coding impact | ClinVar | ACMG classification | MAF | MAF (ref pop) | |
| --- | --- | --- | --- | --- | --- | --- | --- | --- | --- | --- | --- | --- | --- | --- | --- |
| 4 | nCHH | yes | *CHD7* | Het | AD | [rs1244745262](https://varsome.com/variant/hg38/rs1244745262?&annotation-mode=germline) | NM_017780.4 | c.5713A>G | N1905D (p.Asn1905Asp) | missense | nd | Likely benign | 0.00000657 | 0.0000147 |  |
| 2 | nCHH | no | *HESX1* | Het | AD/AR | [rs143057250](https://varsome.com/variant/hg38/rs143057250?&annotation-mode=germline&zygosity=Homozygous&family-members-also-affected=False) | NM_003865.3 | c.385G>A | V129I (p.Val129Ile) | missense | Conflicting | Likely benign | 0.00081 | 0.00133 |  |
| 3 | nCHH | yes* | *SEMA3A* | Het | AD | rs766918295 | NM_006080.3 | c.1910G>A | R637H (p.Arg637His) | missense | Uncertain significance | Likely benign | 0.0000279 | 0.000044 |  |
| 5 | nCHH | no | *SEMA7A* | Het | AD | [rs372161007](https://varsome.com/variant/hg38/rs372161007?) | NM_003612.5 | c.370C>T | R124W (p.Arg124Trp) | missense | nd | Benign | 0.0000641 | 0.0000886 |  |
| 6 | nCHH | no | *SEMA3A* | CH (trans) | AD | [rs139295139](https://varsome.com/variant/hg38/rs139295139?&annotation-mode=germline&zygosity=Homozygous&sex=M&family-members-also-affected=False) | NM_006080.3 | c.458A>G | N153S (p.Asn153Ser) | missense | Conflicting | Benign | 0.00231 | 0.00373 |  |
| 6 | nCHH | no | *SEMA3A* | CH (trans) | AD | nd | NM_006080.3 | c.334-1G>A | - | non coding | nd | Likely pathogenic | nd | nd |  |
| 7 | nCHH | no | *FEZF1* | Het | AD/AR | [rs145467198](https://varsome.com/variant/hg38/rs145467198?&annotation-mode=germline&zygosity=Heterozygous&sex=M&family-members-also-affected=False) | NM_001024613.4 | c.253A>G | S85G (p.Ser85Gly) | missense | Benign | Benign | 0.00582 | 0.00807 |  |
| 8 | nCHH | no | *HS6ST1* | Het | AD | [rs200268730](https://varsome.com/variant/hg38/rs200268730?&annotation-mode=germline&zygosity=Heterozygous&sex=M&family-members-also-affected=False) | NM_004807.3 | c.652C>T | P218S (p.Pro218Ser) | missense | Conflicting | Benign | 0.00183 | 0.00313 |  |
| 17 | nCHH | no | *PROKR2* | Het | AD/AR | [rs368732206](https://varsome.com/variant/hg38/rs368732206?&annotation-mode=germline&zygosity=Heterozygous&sex=M&family-members-also-affected=False) | NM_144773.4 | c.472G>A | V158I (p.Val158Ile) | missense | nd | Benign | 0.00012 | 0.0000972 |  |
| 17 | nCHH | no | *CHD7* | Het | AD | nd | NM_017780.4 | c.4012G>A | G1338S (p.Gly1338Ser) | missense | nd | Likely pathogenic | nd | nd |  |
| 25 | nCHH | no* | *CHD7* | Het | AD | nd | NM_017780.4 | c.2272C>T | R758C (p.Arg758Cys) | missense | nd | Uncertain significance | nd | nd |  |
| 26 | nCHH | no | *PROKR2* | Het | AD/AR | [rs141090506](https://varsome.com/variant/hg38/rs141090506?&annotation-mode=germline&zygosity=Heterozygous&sex=M&family-members-also-affected=False) | NM_144773.4 | c.253C>T | R85C (p.Arg85Cys) | missense | Conflicting | Likely benign | 0.000592 | 0.00142 |  |
| 30 | nCHH | yes* | *HESX1* | Het | AD/AR | rs148422263 | NM_003865.3 | c.220G>A | V74M (p.Val74Met) | missense | Uncertain significance | Likely benign | 0.000111 | 0.000211 |  |
| 30 | nCHH | yes* | *HESX1* | Het | AD/AR | [rs148422263](https://varsome.com/variant/hg38/rs148422263?&annotation-mode=germline&zygosity=Heterozygous&sex=M&inheritance=Father&family-members-also-affected=True&family-segregation=False) | NM_003865.3 | c.220G>A | V74M (p.Val74Met) | missense | Uncertain significance | Likely benign | 0.000111 | 0.000211 |  |
| 32 | nCHH | no | *KISS1R* | Hom | AR | nd | NM_032551.5 | c.346A>G | K116E (p.Lys116Glu) | missense | nd | Uncertain significance | nd | nd |  |
| 41 | nCHH | no* | *SEMA3A* | Het | AD | rs147436181 | NM_006080.3 | c.1303G>A | V435I (p.Val435Ile) | missense | Benign | Benign | 0.0136 | 0.0215 |  |
| 42 | nCHH | no | *SEMA3E* | Het | AD | [rs774452522](https://varsome.com/variant/hg38/rs774452522?&annotation-mode=germline&zygosity=Heterozygous&sex=M&family-members-also-affected=False) | NM_012431.3 | c.2041A>G | M681V (p.Met681Val) | missense | nd | Likely benign | 0.00000799 | 0.0000177 |  |
| 49 | nCHH | no* | *PROK2* | Het | AD/AR | rs1467583182 | NM_001126128.2 | c.92C>T | T31I (p.Thr31Ile) | missense | nd | Uncertain significance | nd | nd |  |
| 50 | nCHH | no* | *GNRHR* | CH (trans) | AR | [rs104893843](https://varsome.com/variant/hg38/rs104893843?) | NM_000406.3 | c.30T>A | N10K (p.Asn10Lys) | missense | Likely pathogenic | Likely pathogenic | 0.000129 | 0.000229 |  |
| 50 | nCHH | no* | *GNRHR* | CH (trans) | AR | [rs104893836](https://varsome.com/variant/hg38/rs104893836?&annotation-mode=germline&zygosity=Homozygous&inheritance=Mother&family-members-also-affected=False&family-segregation=False) | NM_000406.3 | c.317A>G | Q106R (p.Gln106Arg) | missense | Pathogenic | Pathogenic | 0.00284 | 0.00418 |  |
| 50 | nCHH | no* | *GNRHR* | CH (trans) | AR | [rs776834867](https://varsome.com/variant/hg38/rs776834867?&annotation-mode=germline&zygosity=Homozygous&inheritance=Father&family-members-also-affected=False&family-segregation=False) | NM_000406.3 | c.31C>A | Q11K (p.Gln11Lys) | missense | Likely pathogenic | Uncertain significance | 0.000129 | 0.000229 |  |
| 52 | nCHH | no | *SEMA3A* | Het | AD | rs766918295 | NM_006080.3 | c.1910G>A | R637H (p.Arg637His) | missense | Uncertain significance | Likely Benign | 0.0000279 | 0.000044 |  |
| 54 | nCHH | no | *FGFR1* | Het | AD | nd | NM_023110.3 | c.2231G>C | R744T (p.Arg744Thr) | missense | nd | Likely pathogenic | nd | nd |  |
| 58 | nCHH | yes* | *FGFR1* | Het | AD | rs17182463 | NM_023110.3 | c.2464C>T | R822C (p.Arg822Cys) | missense | Likely benign | Benign | 0.000265 | 0.00026 |  |
| 60 | nCHH | no* | *PROK2* | Het | AD/AR | nd | NM_001126128.2 | c.2T>G | M1R (p.Met1Arg) | start loss | nd | Likely pathogenic | nd | nd |  |
| 65 | nCHH | no* | *FLRT3* | Het | AD | rs36034779 | NM_198391.3 | c.1134A>C | Q378H (p.Gln378His) | missense | Likely benign | Benign | 0.018 | 0.0307 |  |
| 68 | nCHH | no | *GNRHR* | CH (trans) | AR | nd | NM_000406.3 | c.257C>T | A86V (p.Ala86Val) | missense | nd | Uncertain significance | nd | nd |  |
| 68 | nCHH | no | *GNRHR* | CH (trans) | AR | nd | NM_000406.3 | c.256G>C | A86P (p.Ala86Pro) | missense | nd | Uncertain significance | nd | nd |  |
| 71 | nCHH | no* | *SOX10* | Het | AD | nd | NM_006941.4 | c.1039C>A | P347T (p.Pro347Thr) | missense | nd | Uncertain significance | nd | nd |  |
| 75 | nCHH | no | *IL17RD* | Het | AR | [rs201420445](https://varsome.com/variant/hg38/rs201420445?&annotation-mode=germline&zygosity=Heterozygous&sex=M&family-members-also-affected=False&family-segregation=False) | NM_017563.5 | c.1946C>T | T649M (p.Thr649Met) | missense | Uncertain significance | Likely benign | 0.000192 | 0.000416 |  |
| 78 | nCHH | no | *GNRH1* | Het | AR | nd | NM_000825.3 | c.53T>G | L18R (p.Leu18Arg) | missense | nd | Uncertain significance | nd | nd |  |
| 79 | nCHH | no* | *GNRHR* | Het | AR | [rs104893836](https://varsome.com/variant/hg38/rs104893836?&annotation-mode=germline&zygosity=Heterozygous&sex=M&inheritance=Father&family-members-also-affected=False&family-segregation=False) | NM_000406.3 | c.317A>G | Q106R (p.Gln106Arg) | missense | Pathogenic | Pathogenic | 0.00284 | 0.00418 |  |
| 80 | nCHH | no | *PROKR2* | Het | AD/AR | rs141090506 | NM_144773.4 | c.253C>T | R85C (p.Arg85Cys) | missense | Conflicting | Likely Benign | 0.000592 | 0.000369 |  |
| 81 | nCHH | no | *NELF* | Het | AD | rs760545169 | NM_015537.5 | c.842G>A | R281H (p.Arg281His) | missense | nd | Likely Benign | 0.0000183 | nd |  |
| 82 | nCHH | no* | *FGFR1* | Het | AD | nd | NM_023110.3 | c.2148delT | H717Tfs*15 (p.His717ThrfsTer15) | frameshift | nd | Likely Pathogenic | nd | nd |  |
| 83 | nCHH | no* | *FGF8* | Het | AD | rs548987968 | NM_033163.5 | c.583C>T | R195W (p.Arg195Trp) | missense | nd | Likely Benign | 0.000012 | 0.00000886 |  |
| 85 | nCHH | yes | *CHD7* | Het | AD | rs772257683 | NM_017780.4 | c.830C>T | P277L (p.Pro277Leu) | missense | nd | Likely Benign | 0.0000522 | 0.0000532 |  |
| 87 | nCHH | no | *KISS1R* | Het | AR | rs371771794 | NM_032551.5 | c.1167C>A | C389* (p.Cys389Ter) | nonsense | Uncertain Significance | Uncertain Significance | 0.000355 | 0.000481 |  |
| 89 | nCHH | no* | *FGFR1* | Het | AD | rs770139002 | NM_023110.3 | c.584A>G | K195R (p.Lys195Arg) | missense | Uncertain Significance | Uncertain Significance | 0.000016 | nd |  |
| 93 | nCHH | no* | *FGFR1* | Het | AD | nd | NM_023110.3 | c.1141dupT | C381Lfs*27 (p.Cys381LeufsTer27) | frameshift | nd | Likely pathogenic | nd | nd |  |
| 94 | nCHH | no | *GNRHR* | Hom | AR | [rs104893836](https://varsome.com/variant/hg38/rs104893836?&annotation-mode=germline&zygosity=Homozygous&inheritance=Mother&family-members-also-affected=False&family-segregation=False) | NM_000406.3 | c.317A>G | Q106R (p.Gln106Arg) | missense | Pathogenic | Pathogenic | 0.00284 | 0.00418 |  |
| 94 | nCHH | no* | *SPRY4* | Het | AD | rs139512218 | NM_030964.5 | c.722C>T | S241F (p.Ser241Phe) | missense | Conflicting | Uncertain significance | 0.00446 | 0.00611 |  |
| 99 | nCHH | yes* | *FGFR1* | Het | AD | nd | NM_023110.3 | c.1657C>T | Q553* (p.Gln553Ter) | nonsense | nd | Likely pathogenic | nd | nd |  |
| 101 | nCHH | no | *PROKR2* | Het | AD/AR | [rs74315416](https://varsome.com/variant/hg38/rs74315416?&annotation-mode=germline) | NM_144773.4 | c.518T>G | L173R (p.Leu173Arg) | missense | Conflicting | Likely pathogenic | 0.00212 | 0.00347 |  |
| 101 | nCHH | no | *CHD7* | Het | AD | nd | NM_017780.4 | c.4012G>A | G1338S (p.Gly1338Ser) | missense | nd | Likely pathogenic | nd | nd |  |
| 104 | nCHH | yes | *FGFR1* | Het | AD | nd | NM_023110.3 | c.418_419delGA | E140Nfs*3 (p.Glu140AsnfsTer3) | frameshift | nd | Likely pathogenic | nd | nd |  |
| 105 | nCHH | no | *PROKR2* | Het | AD/AR | [rs368732206](https://varsome.com/variant/hg38/rs368732206?&annotation-mode=germline&zygosity=Heterozygous&sex=M&family-members-also-affected=False) | NM_144773.4 | c.472G>A | V158I (p.Val158Ile) | missense | nd | Benign | 0.00012 | 0.0000972 |  |
| 105 | nCHH | no | *TACR3* | Het | AD/AR | [rs780364097](https://varsome.com/variant/hg38/rs780364097?&annotation-mode=germline&zygosity=Heterozygous&sex=M&family-members-also-affected=False) | NM_001059.3 | c.437C>T | A146V (p.Ala146Val) | missense | nd | Uncertain significance | 0.00000398 | 0.00000879 |  |
| 110 | nCHH | yes | *TACR3* | Het | AD/AR | nd | NM_001059.3 | c.238+1G>C | - | non coding | nd | Uncertain significance | nd | nd |  |
| 110 | nCHH | yes | *FLRT3* | Het | AD | [rs36034779](https://varsome.com/variant/hg38/rs36034779?&annotation-mode=germline&zygosity=Heterozygous&sex=M&family-members-also-affected=True) | NM_198391.3 | c.1134A>C | Q378H (p.Gln378His) | missense | Likely benign | Benign | 0.018 | 0.0307 |  |
| 111 | nCHH | no | *FGFR1* | Het | AD | rs727505376 | NM_023110.3 | c.2059G>A | G687R (p.Gly687Arg) | missense | Pathogenic | Pathogenic | nd | nd |  |
| 113 | nCHH | yes | *SPRY4* | Het | AD | rs770167713 | NM_030964.5 | c.502G>T | G168C (p.Gly168Cys) | missense | nd | Uncertain significance | 0.0000119 | 0.0000264 |  |
| 117 | nCHH | no | *PROKR2* | Het | AD/AR | rs117106081 | NM_144773.4 | c.991G>A | V331M (p.Val331Met) | missense | Likely Benign | Benign | 0.0062 | 0.000308 |  |
| 118 | nCHH | yes | *GNRH1* | Het | AR | rs1336171284 | NM_000825.3 | c.104G>A | R35H (p.Arg35His) | missense | Pathogenic | Likely Pathogenic | 0.00000401 | 0.00000884 |  |
| 120 | nCHH | no* | *PROKR2* | Het | AD/AR | rs587777834 | NM_144773.4 | c.58delC | H20Mfs*24 (p.His20MetfsTer24) | frameshift | Conflicting | Pathogenic | 0.000103 | 0.000176 |  |
| 120 | nCHH | no* | *HESX1* | CH (cis) | AD/AR | rs1481713480 | NM_003865.3 | c.531A>C | K177N (p.Lys177Asn) | missense | nd | Likely Benign | 0.00000657 | 0.0000147 |  |
| 120 | nCHH | no* | *HESX1* | CH (cis) | AD/AR | rs759180706 | NM_003865.3 | c.533delA | N178Ifs*10 (p.Asn178IlefsTer10) | framshift | nd | Uncertain significance | 0.000016 | 0.00000884 |  |
| 122 | nCHH | no | *PROKR2* | Het | AD/AR | rs199696443 | NM_144773.4 | c.916G>A | V306M (p.Val306Met) | missense | nd | Benign | 0.0000239 | 0.0000264 |  |
| 122 | nCHH | no | *KISS1R* | Het | AR | nd | NM_032551.5 | c.424T>G | Y142D (p.Tyr142Asp) | missense | nd | Uncertain significance | nd | nd |  |
| 130 | nCHH | yes* | *PROKR2* | Het | AD/AR | [rs74315416](https://varsome.com/variant/hg38/rs74315416?&annotation-mode=germline) | NM_144773.4 | c.518T>G | L173R (p.Leu173Arg) | missense | Conflicting | Likely pathogenic | 0.00212 | 0.00347 |  |
| 133 | nCHH | no | *FGFR1* | Het | AD | rs727505373 | NM_023110.3 | c.296A>G | Y99C (p.Tyr99Cys) | missense | Likely Pathogenic | Pathogenic | nd | nd |  |
| 139 | nCHH | no | *SEMA7A* | Het | AD | rs143841629 | NM_003612.5 | c.1864C>T | R622C (p.Arg622Cys) | missense | nd | Benign | 0.000589 | 0.000414 |  |
| 143 | nCHH | yes* | *TACR3* | Het | AD/AR | nd | NM_001059.3 | c.799T>C | Y267H (p.Tyr267His) | missense | nd | Uncertain significance | nd | nd |  |
| 143 | nCHH | yes* | *CHD7* | Het | AD | rs200188105 | NM_017780.4 | c.2675G>A | R892H (p.Arg892His) | missense | Likely Benign | Benign | 0.000139 | 0.0000361 |  |
| 145 | nCHH | no | *PROK2* | Het | AD/AR | rs144953748 | NM_001126128.2 | c.301C>T | R101W (p.Arg101Trp) | missense | Uncertain significance | Benign | 0.000127 | 0.0000704 |  |
| 146 | nCHH | yes* | *TACR3* | Hom | AD/AR | rs144292455 | NM_001059.3 | c.824G>A | W275* (p.Trp275Ter) | nonsense | Pathogenic | Pathogenic | 0.000307 | 0.000626 |  |
| 146 | nCHH | yes* | *CHD7* | Het | AD | rs772369092 | NM_017780.4 | c.1315C>T | P439S (p.Pro439Ser) | missense | Uncertain significance | Uncertain significance | 0.00000401 | 0.00000885 |  |
| 153 | nCHH | no | *SEMA3A* | Het | AD | rs778581008 | NM_006080.3 | c.1717+3A>G | - | noncoding | nd | uncertain significance | 0.00000455 | 0.00000969 |  |
| 162 | nCHH | no* | *KISS1* | Het | AD | rs748737279 | NM_002256.4 | c.76G>A | A26T (p.Ala26Thr) | missense | nd | Likely Benign | 0.0000657 | 0.0000198 |  |
| 172 | nCHH | no | *KISS1R* | Hom | AR | [rs104894702](https://varsome.com/variant/hg38/rs104894702?&annotation-mode=germline&zygosity=Homozygous&family-members-also-affected=True) | NM_032551.5 | c.1195T>A | *399Rext* (p.Ter399Argext*) | stopLoss | Likely pathogenetic | Pathogenic | 0.0000681 | 0.000112 |  |
| 172 | nCHH | no* | *CHD7* | Het | AD | nd | NM_017780.4 | c.218A>G | N73S (p.Asn73Ser) | missense | nd | Likely Benign | nd | nd |  |
| 174 | nCHH | no* | *KISS1R* | Hom | AR | rs104894703 | NM_032551.5 | c.305T>C | L102P (p.Leu102Pro) | missense | Pathogenic | Likely Pathogenic | 0.0000255 | 0.0000331 |  |
| 177 | KS | no* | *FGFR1* | Het | AD | nd | NM_023110.3 | c.1553-2G>A | - | non coding | nd | Likely Pathogenic | nd | nd |  |
| 178 | KS | no* | *SEMA3A* | Het | AD | [rs199979628](https://varsome.com/variant/hg38/rs199979628?&annotation-mode=germline&zygosity=Heterozygous&sex=M&inheritance=Mother&family-members-also-affected=False&family-segregation=False) | NM_006080.3 | c.196C>T | R66W (p.Arg66Trp) | missense | Uncertain significance | Likely pathogenic | 0.000525 | 0.000819 |  |
| 180 | KS | no | *FGFR1* | Het | AD | nd | NM_023110.3 | c.888T>A | N296K (p.Asn296Lys) | missense | nd | Likely pathogenic | nd | nd |  |
| 180 | KS | no | *SEMA3A* | Het | AD | [rs139295139](https://varsome.com/variant/hg38/rs139295139?&annotation-mode=germline&zygosity=Heterozygous&sex=M&family-members-also-affected=False) | NM_006080.3 | c.458A>G | N153S (p.Asn153Ser) | missense | Conflicting | Benign | 0.00231 | 0.00373 |  |
| 186 | KS | no | *FEZF1* | Het | AD/AR | [rs145467198](https://varsome.com/variant/hg38/rs145467198?&annotation-mode=germline&zygosity=Heterozygous&sex=M&family-members-also-affected=False) | NM_001024613.4 | c.253A>G | S85G (p.Ser85Gly) | missense | Benign | Benign | 0.00582 | 0.00807 |  |
| 190 | KS | yes* | *WDR11* | Het | AD | [rs61761620](https://varsome.com/variant/hg38/rs61761620?&annotation-mode=germline&zygosity=Heterozygous&sex=M&inheritance=Mother&family-members-also-affected=True&family-segregation=False) | NM_018117.12 | c.2305A>G | M769V (p.Met769Val) | missense | Uncertain significance | Benign | 0.000565 | 0.000202 |  |
| 193 | KS |  | *FGF8* | Het | AD | [rs137852660](https://varsome.com/variant/hg38/rs137852660?&annotation-mode=germline&zygosity=Heterozygous&sex=M) | NM_033163.5 | c.77C>T | P26L (p.Pro26Leu) | missense | Conflicting | Uncertain significance | 0.00115 | 0.00119 |  |
| 194 | KS | yes* | *SEMA3A* | Het | AD | [rs139295139](https://varsome.com/variant/hg38/rs139295139?&annotation-mode=germline&zygosity=Heterozygous&sex=M&inheritance=Father&family-members-also-affected=True&family-segregation=False) | NM_006080.3 | c.458A>G | N153S (p.Asn153Ser) | missense | Conflicting | Benign | 0.00231 | 0.00373 |  |
| 194 | KS | yes* | *FGF8* | Het | AD | [rs771618872](https://varsome.com/variant/hg38/rs771618872?&annotation-mode=germline&zygosity=Heterozygous&sex=M&inheritance=Father&family-members-also-affected=True&family-segregation=False) | NM_033163.5 | c.98G>T | G33V (p.Gly33Val) | missense | nd | Benign | 0.0000271 | 0.0000543 |  |
| 196 | KS | no | *SOX10* | Het | AD | nd | NM_006941.4 | c.7G>T | E3* (p.Glu3Ter) | nonsense | Pathogenic | Pathogenic | nd | nd |  |
| 200 | KS | no | *CHD7* | Het | AD |  | NM_017780.4 | c.2442+5G>A | - | non coding | nd | Uncertain significance | nd | nd |  |
| 201 | KS | no* | *ANOS1* | Hem | XLinked | nd | NM_000216.4 | c.226delT | W76Gfs*21 (p.Trp76GlyfsTer21) | framshift | nd | Likely pathogenic | nd | nd |  |
| 201 | KS | no* | *SEMA7A* | Het | AD | [rs1239642047](https://varsome.com/variant/hg38/rs1239642047?&annotation-mode=germline&zygosity=Heterozygous&sex=M&family-members-also-affected=False) | NM_003612.5 | c.1462G>C | V488L (p.Val488Leu) | missense | nd | Likely benign | 0.000004 | 0.00000887 |  |
| 205 | KS | no | *ANOS1* | Hem | XLinked | nd | NM_000216.4 | c.1759G>C | V587L (p.Val587Leu) | missense | nd | Pathogenic | nd | nd |  |
| 207 | KS | no* | *PROKR2* | Het | AD/AR | [rs74315416](https://varsome.com/variant/hg38/rs74315416?&annotation-mode=germline) | NM_144773.4 | c.518T>G | L173R (p.Leu173Arg) | missense | Conflicting | Likely pathogenic | 0.00212 | 0.00347 |  |
| 207 | KS | no* | *GNRHR* | Het | AR | [rs104893836](https://varsome.com/variant/hg38/rs104893836?&annotation-mode=germline&zygosity=Heterozygous&sex=M&inheritance=Mother&family-members-also-affected=False&family-segregation=False) | NM_000406.3 | c.317A>G | Q106R (p.Gln106Arg) | missense | Pathogenic | Pathogenic | 0.00284 | 0.00418 |  |
| 209 | KS | yes* | *PROKR2* | Het | AD/AR | [rs74315416](https://varsome.com/variant/hg38/rs74315416?&annotation-mode=germline) | NM_144773.4 | c.518T>G | L173R (p.Leu173Arg) | missense | Conflicting | Likely pathogenic | 0.00212 | 0.00347 |  |
| 209 | KS | yes* | *TACR3* | Het | AD/AR | [rs17033889](https://varsome.com/variant/hg38/rs17033889?&annotation-mode=germline&zygosity=Heterozygous&sex=M&inheritance=Mother&family-members-also-affected=True&family-segregation=False) | NM_001059.3 | c.1345G>A | A449T (p.Ala449Thr) | missense | Benign | Benign | 0.00532 | 0.00707 |  |
| 210 | KS | yes | *FGFR1* | Het | AD | nd | NM_023110.3 | c.1368G>A | M456I (p.Met456Ile) | missense | nd | Uncertain significance | nd | nd |  |
| 210 | KS | yes | *TACR3* | Het | AD/AR | [rs202051644](https://varsome.com/variant/hg38/rs202051644?&annotation-mode=germline&zygosity=Heterozygous&sex=M&family-members-also-affected=True) | NM_001059.3 | c.1274G>A | R425Q (p.Arg425Gln) | missense | nd | Likely benign | 0.0000517 | 0.0000793 |  |
| 213 | KS | no | *HS6ST1* | Het | AD | nd | NM_004807.3 | c.955T>A | Y319N (p.Tyr319Asn) | missense | Uncertain significance | Uncertain significance | nd | nd |  |
| 216 | KS | yes | *ANOS1* | Hem | XLinked | nd | NM_000216.4 | c.571C>T | R191* (p.Arg191Ter) | nonsense | Pathogenic | Pathogenic | nd | nd |  |
| 217 | KS | no | *FGFR1* | Het | AD | nd | NM_023110.3 | c.1058C>G | S353C (p.Ser353Cys) | missense | nd | Uncertain significance | nd | nd |  |
| 219 | KS | yes | *PROKR2* | Hom | AD/AR | [rs141090506](https://varsome.com/variant/hg38/rs141090506?&annotation-mode=germline&zygosity=Homozygous&sex=M&family-members-also-affected=True) | NM_144773.4 | c.253C>T | R85C (p.Arg85Cys) | missense | Conflicting | Likely benign | 0.000592 | 0.000369 |  |
| 220 | KS | yes* | *FGFR1* | Het | AD | nd | NM_023110.3 | c.290G>T | G97V (p.Gly97Val) | missense | nd | Pathogenic | nd | nd |  |
| 221 | KS | yes* | *KISS1R* | Het | AR | [rs765739273](https://varsome.com/variant/hg38/rs765739273?&annotation-mode=germline&zygosity=Heterozygous&sex=M&inheritance=Mother&family-members-also-affected=True&family-segregation=False) | NM_032551.5 | c.407T>G | M136R (p.Met136Arg) | missense | nd | Likely benign | 0.00000564 | 0.0000131 |  |
| 221 | KS | yes* | *CHD7* | Het | AD | nd | NM_017780.4 | c.4275C>G | F1425L (p.Phe1425Leu) | missense | nd | Uncertain significance | nd | nd |  |
| 223 | KS | no | *FGFR1* | Het | AD | [rs781310679](https://varsome.com/variant/hg38/rs781310679?&annotation-mode=germline&zygosity=Heterozygous&sex=M&family-members-also-affected=False) | NM_023110.3 | c.1408C>T | R470C (p.Arg470Cys) | missense | Uncertain significance | Likely benign | 0.0000721 | 0.0000441 |  |
| 225 | KS | no | *IL17RD* | Het | AR | [rs140930246](https://varsome.com/variant/hg38/rs140930246?&annotation-mode=germline&zygosity=Heterozygous&sex=M&family-members-also-affected=False) | NM_017563.5 | c.1972A>G | M658V (p.Met658Val) | missense | Likely benign | Likely benign | 0.00106 | nd |  |
| 226 | KS | no* | *ANOS1* | Hem | XLinked | nd | NM_000216.4 | c.814C>T | R272* (p.Arg272Ter) | nonsense | Pathogenic | Pathogenic | nd | nd |  |
| 230 | KS | no | *PROKR2* | Het | AD/AR | [rs78861628](https://varsome.com/variant/hg38/rs78861628?&annotation-mode=germline&zygosity=Heterozygous&sex=M&family-members-also-affected=False) | NM_144773.4 | c.802C>T | R268C (p.Arg268Cys) | missense | Likely benign | Benign | 0.00391 | 0.000449 |  |
| 230 | KS | no | *NELF* | Het | AD | rs373616272 | NM_015537.5 | c.1487A>T | Q496L (p.Gln496Leu) | missense | nd | Benign | 0.0000282 | nd |  |
| 231 | KS | yes* | *PROKR2* | Het | AD/AR | [rs781065670](https://varsome.com/variant/hg38/rs781065670?&annotation-mode=germline&zygosity=Heterozygous&sex=M&inheritance=Mother&family-members-also-affected=True&family-segregation=False) | NM_144773.4 | c.803G>A | R268H (p.Arg268His) | missense | Uncertain significance | Benign | 0.000119 | 0.000185 |  |
| 232 | KS | yes* | *ANOS1* | Hem | XLinked | rs749306310 | NM_000216.4 | c.1984+2T>A | - | non coding | nd | Likely Pathogenic | 0.000011 | 0.0000247 |  |
| 233 | KS | yes* | *ANOS1* | Hem | XLinked | rs749306310 | NM_000216.4 | c.1984+2T>A | - | non coding | nd | Likely Pathogenic | 0.000011 | 0.0000247 |  |
| 234 | KS | yes* | *ANOS1* | Hem | XLinked | nd | NM_000216.4 | c.878delC | P293Rfs*17 (p.Pro293ArgfsTer17) | frameshift | nd | Likely Pathogenic | nd | nd |  |
| 236 | KS | no* | *PROKR2* | Het | AD/AR | [rs74315416](https://varsome.com/variant/hg38/rs74315416?&annotation-mode=germline) | NM_144773.4 | c.518T>G | L173R (p.Leu173Arg) | missense | Conflicting | Likely pathogenic | 0.00212 | 0.00347 |  |
| 236 | KS | no* | *FEZF1* | Het | AD/AR | nd | NM_001024613.4 | c.382A>C | K128Q (p.Lys128Gln) | missense | nd | Likely benign | nd | nd |  |
| 240 | KS | no | *PROKR2* | Het | AD/AR | rs779246131 | NM_144773.4 | c.1145G>A | R382K (p.Arg382Lys) | missense | nd | Likely benign | 0.00000798 | 0.0000177 |  |
| 240 | KS | no | *CHD7* | Het | AD | rs202039728 | NM_017780.4 | c.8672A>G | N2891S (p.Asn2891Ser) | missense | Conflicting | Benign | 0.000233 | 0.000336 |  |
| 241 | KS | no | *FGFR1* | Het | AD | [rs121909645](https://varsome.com/variant/hg38/rs121909645?&annotation-mode=germline&zygosity=Heterozygous&sex=M&family-members-also-affected=False) | NM_023110.3 | c.749G>A | R250Q (p.Arg250Gln) | missense | Risk factor | Pathogenic | nd | nd |  |
| 243 | KS | yes* | *ANOS1* | Hem | XLinked | nd | NM_000216.4 | c.545delT | V182Afs*3 (p.Val182AlafsTer3) | frameshift | nd | Likely pathogenic | nd | nd |  |
| 244 | KS | yes | *SEMA3A* | Het | AD | [rs147436181](https://varsome.com/variant/hg38/rs147436181?&annotation-mode=germline&zygosity=Heterozygous&sex=M&family-members-also-affected=True) | NM_006080.3 | c.1303G>A | V435I (p.Val435Ile) | missense | Benign | Benign | 0.0136 | 0.0215 |  |
| 247 | KS | yes | *PROK2* | Het | AD/AR | [rs756449447](https://varsome.com/variant/hg38/rs756449447?&annotation-mode=germline&zygosity=Heterozygous&sex=M&family-members-also-affected=True) | NM_001126128.2 | c.146G>T | S49I (p.Ser49Ile) | missense | nd | Uncertain significance | 0.00000398 | nd |  |
| 252 | KS | no | *ANOS1* | Hem | XLinked | nd | NM_000216.4 | c.1376_1377delAT | H459Rfs*6 (p.His459ArgfsTer6) | frameshift | nd | Likely pathogenic | nd | nd |  |
| 253 | KS | no | *PROKR2* | Het | AD/AR | rs149396342 | NM_144773.4 | c.403C>T | R135C (p.Arg135Cys) | missense | Uncertain significance | Likely benign | 0.0000477 | 0.0000704 |  |
| 253 | KS | no | *SPRY4* | Het | AD | [rs148983803](https://varsome.com/variant/hg38/rs148983803?&annotation-mode=germline&zygosity=Heterozygous&sex=M&family-members-also-affected=False) | NM_030964.5 | c.626G>A | C209Y (p.Cys209Tyr) | missense | Likely benign | Uncertain significance | 0.00217 | 0.0035 |  |
| 253 | KS | no | *SEMA3A* | Het | AD | [rs147436181](https://varsome.com/variant/hg38/rs147436181?&annotation-mode=germline&zygosity=Heterozygous&sex=M&family-members-also-affected=False) | NM_006080.3 | c.1303G>A | V435I (p.Val435Ile) | missense | Benign | Benign | 0.0136 | 0.0215 |  |
| 255 | KS | no | *PROKR2* | Het | AD/AR | [rs74315416](https://varsome.com/variant/hg38/rs74315416?&annotation-mode=germline) | NM_144773.4 | c.518T>G | L173R (p.Leu173Arg) | missense | Conflicting | Likely pathogenic | 0.00212 | 0.00347 |  |
| 257 | KS | no | *GNRHR* | Het | AR | rs104893836 | NM_000406.3 | c.317A>G | Q106R (p.Gln106Arg) | missense | Pathogenic | Pathogenic | 0.00284 | 0.00418 |  |
| 258 | KS | no* | *CHD7* | CH | AD | rs753953205 | NM_017780.4 | c.1375C>T | R459C (p.Arg459Cys) | missense | Conflicting | Benign | 0.000189 | 0.0000621 |  |
| 258 | KS | no* | *CHD7* | CH | AD | nd | NM_017780.4 | c.5945G>A | G1982E (p.Gly1982Glu) | missense | nd | Likely Pathogenic | nd | nd |  |
| 259 | KS | no* | *HESX1* | Het | AD/AR | [rs28936704](https://varsome.com/variant/hg38/rs28936704?&annotation-mode=germline&zygosity=Heterozygous&sex=M&inheritance=Mother&family-members-also-affected=False&family-segregation=False) | NM_003865.3 | c.541A>G | T181A (p.Thr181Ala) | missense | Uncertain significance | Uncertain significance | 0.000104 | 0.000212 |  |
| 260 | KS | yes* | *FGFR1* | Het | AD | nd | NM_023110.3 | c.263T>G | V88G (p.Val88Gly) | missense | nd | Uncertain significance | nd | nd |  |
| 260 | KS | yes* | *FEZF1* | Het | AD/AR | rs145467198 | NM_001024613.4 | c.253A>G | S85G (p.Ser85Gly) | missense | Benign | Benign | 0.00582 | 0.00807 |  |
| 260 | KS | yes* | *GNRHR* | Het | AR | rs104893836 | NM_000406.3 | c.317A>G | Q106R (p.Gln106Arg) | missense | Pathogenic | Pathogenic | 0.00284 | 0.00418 |  |
| 261 | KS | no | *SEMA7A* | Het | AD | rs143621767 | NM_003612.5 | c.1085G>A | R362Q (p.Arg362Gln) | missense | nd | Benign | 0.0000915 | 0.000132 |  |
| 264 | KS | no | *HESX1* | Het | AD/AR | rs148422263 | NM_003865.3 | c.220G>A | V74M (p.Val74Met) | missense | Uncertain significance | Likely benign | 0.000111 | 0.000211 |  |
| 268 | KS | no | *FGFR1* | Het | AD | rs575766741 | NM_023110.3 | c.1363C>G | P455A (p.Pro455Ala) | missense | nd | Uncertain significance | 0.000012 | 0.00000883 |  |
| 269 | KS | no | *CHD7* | Het | AD | rs757707495 | NM_017780.4 | c.8188G>A | A2730T (p.Ala2730Thr) | missense | Uncertain significance | Uncertain significance | 0.0000125 | 0.0000183 |  |
| 270 | KS | no* | *PROKR2* | Het | AD/AR | rs141090506 | NM_144773.4 | c.253C>T | R85C (p.Arg85Cys) | missense | Conflicting | Likely Benign | 0.000592 | 0.000369 |  |
| 270 | KS | no* | *PROK2* | Het | AD/AR | nd | NM_001126128.2 | c.137G>T | C45F (p.Cys45Phe) | missense | nd | Uncertain significance | nd | nd |  |
| 270 | KS | no* | *SEMA3A* | Het | AD | rs147436181 | NM_006080.3 | c.1303G>A | V435I (p.Val435Ile) | missense | Benign | Benign | 0.0136 | 0.0215 |  |
| 271 | KS | no | *SOX10* | Het | AD | rs61756177 | NM_006941.4 | c.601G>A | A201T (p.Ala201Thr) | missense | nd | Likely Benign | 0.0000243 | 0.000027 |  |
| 272 | KS | no | *ANOS1* | Hem | XLinked | nd | NM_000216.4 | c.1903C>T | Q635* (p.Gln635Ter) | nonsense | nd | Likely Pathogenic | nd | nd |  |
| 274 | KS | no | *FEZF1* | Het | AD/AR | rs1454327695 | NM_001024613.4 | c.722T>A | I241N (p.Ile241Asn) | missense | nd | Uncertain significance | 0.00000398 | 0.00000879 |  |
| 275 | KS | yes* | *ANOS1* | Hem | XLinked | rs1287903806 | NM_000216.4 | c.1984+4A>G | - | nd | nd | Uncertain significance | nd | nd |  |
| 276 | KS | yes | *ANOS1* | Hem | XLinked | nd | NM_000216.4 | c.1468C>T | Q490* (p.Gln490Ter) | nonsense | nd | Likely Pathogenic | nd | nd |  |
| 276 | KS | yes | *SPRY4* | Het | AD | nd | NM_030964.5 | c.622delG | E208Sfs*74 (p.Glu208SerfsTer74) | frameshift | nd | Likely Pathogenic | nd | nd |  |
| 279 | KS | no | *KISS1R* | Het | AR | rs764262625 | NM_032551.5 | c.1041_1049delCCCCCGCCG | R349_P351del (p.Arg349_Pro351del) | in frame | nd | Uncertain significance | 0.000111 | 0.000179 |  |
| 280 | KS | no | *FGF8* | Het | AD | rs137852660 | NM_033163.5 | c.77C>T | P26L (p.Pro26Leu) | missense | Conflicting | Uncertain significance | 0.00115 | 0.00119 |  |
| 284 | KS | no* | *PROKR2* | Het | AD/AR | rs587777834 | NM_144773.4 | c.58delC | H20Mfs*24 (p.His20MetfsTer24) | frameshift | Conflicting | Pathogenic | 0.000103 | 0.000176 |  |
| 285 | KS | no | *PROKR2* | Het | AD/AR | rs144994507 | NM_144773.4 | c.151A>G | A51T (p.Ala51Thr) | missense | Conflicting | Benign | 0.00449 | 0.00222 |  |
| 287 | KS | yes | *SPRY4* | Het | AD | rs78310959 | NM_030964.5 | c.530A>G | K177R (p.Lys177Arg) | missense | Conflicting | Uncertain significance | 0.0016 | 0.00235 |  |
| 292 | KS | yes | *CHD7* | Het | AD | nd | NM_017780.4 | c.6185G>C | R2062P (p.Arg2062Pro) | missense | nd | Uncertain significance | nd | nd |  |
| 294 | KS | no | *SOX10* | Het | AD | nd | NM_006941.4 | c.506C>T | P169L (p.Pro169Leu) | missense | nd | Likely Pathogenic | nd | nd |  |
| 296 | KS | no | *PROK2* | Het | AD/AR | rs554675432 | NM_001126128.2 | c.163del | I55* (p.Ile55Ter) | nonsense | Pathogenic | Pathogenic | 0.000119 | 0.000255 |  |
| 302 | KS | no | *ANOS1* | Hem | XLinked | nd | NM_000216.4 | c.137G>A | R46H (p.Arg46His) | missense | nd | Likely Benign | nd | nd |  |
| 302 | KS | no | *ANOS1* | Het | XLinked | rs45521933 | NM_000216.4 | c.8416C>G | L2806V (p.Leu2806Val) | missense | Likely Benign | Benign | 0.00123 | 0.000689 |  |
| 306 | KS | yes* | *FGFR1* | Het | AD | nd | NM_023110.3 | c.1552+1G>A | - | nd | nd | Likely Pathogenic | nd | nd |  |
| 309 | KS | yes | *FEZF1* | Het | AD/AR | rs1454327695 | NM_001024613.4 | c.722T>A | I241N (p.Ile241Asn) | missense | nd | Uncertain significance | 0.00000398 | 0.00000879 |  |
| 309 | KS | yes | *WDR11* | Het | AD | rs779287029 | NM_018117.12 | c.1649T>C | V550A (p.Val550Ala) | missense | nd | Benign | 0.0000558 | 0.0000264 |  |
| 310 | KS | no* | *CHD7* | CH | AD | nd | NM_017780.4 | c.2182delG | D728Tfs*27 (p.Asp728ThrfsTer27) | frameshift | nd | Likely Pathogenic | nd | nd |  |
| 310 | KS | no* | *CHD7* | CH | AD | rs750444222 | NM_017780.4 | c.2224G>A | D742N (p.Asp742Asn) | missense | nd | Uncertain significance | 0.00000806 | nd |  |
| 311 | KS | no | *ANOS1* | Hem | XLinked | rs1555893221 | NM_000216.4 | c.1267C>T | R423* (p.Arg423Ter) | nonsense | Pathogenic | Pathogenic | nd | nd |  |
| 324 | nCHH | no | *HESX1* | Het | AD/AR | rs121909173 | NM_003865.3 | c.18G>C | Q6H (p.Gln6His) | missense | Uncertain significance | Uncertain significance | 0.0000278 | 0.0000527 |  |
| 326 | nCHH | no | *GNRHR* | Het | AR | rs104893836 | NM_000406.3 | c.317A>G | Q106R (p.Gln106Arg) | missense | Pathogenic | Pathogenic | 0.00284 | 0.00418 |  |
| 326 | nCHH | no | *FLRT3* | Het | AD | nd | NM_198391.3 | c.1859T>G | M620R (p.Met620Arg) | missense | nd | Uncertain significance | nd | nd |  |
| 329 | nCHH | no | *GNRHR* | CH (cis) | AR | rs104893843 | NM_000406.3 | c.30T>A | N10K (p.Asn10Lys) | missense | Likely Pathogenic | Likely Pathogenic | 0.000129 | 0.000229 |  |
| 329 | nCHH | no | *GNRHR* | CH (cis) | AR | rs776834867 | NM_000406.3 | c.31C>A | Q11K (p.Gln11Lys) | missense | Likely Pathogenic | Uncertain significance | 0.000129 | 0.000229 |  |
| 329 | nCHH | no | *GNRHR* | CH (cis) | AR | nd | NM_000406.3 | c.584A>G | Q195R (p.Gln195Arg) | missense | nd | Uncertain significance | nd | nd |  |
| 335 | KS | no | *ANOS1* | Hem | XLinked | nd | NM_000216.4 | c.658C>T | Q220* (p.Gln220Ter) | nonsense | nd | Likely Pathogenic | nd | nd |  |
| 335 | KS | no | *CHD7* | Het | AD | rs202039728 | NM_017780.4 | c.8672A>G | N2891S (p.Asn2891Ser) | missense | Conflicting | Benign | 0.000233 | 0.000336 |  |
| 337 | KS | yes | *FGFR1* | Het | AD | nd | NM_023110.3 | c.533G>A | C178Y (p.Cys178Tyr) | missense | nd | Likely Pathogenic | nd | nd |  |
| 337 | KS | yes | *SEMA7A* | Het | AD | rs768477715 | NM_003612.5 | c.406C>T | R136W (p.Arg136Trp) | missense | nd | Benign | 0.000044 | 0.0000531 |  |
| 339 | KS | yes* | *FGF8* | Het | AD | rs769756528 | NM_033163.5 | c.386G>A | R129Q (p.Arg129Gln) | missense | nd | Likely Benign | 0.0000239 | 0.0000264 |  |
| 339 | KS | yes* | *PROKR2* | Het | AD/AR | [rs74315416](https://varsome.com/variant/hg38/rs74315416?&annotation-mode=germline) | NM_144773.4 | c.518T>G | L173R (p.Leu173Arg) | missense | Conflicting | Likely pathogenic | 0.00212 | 0.00347 |  |
| 344 | KS | no* | *GNRHR* | CH (trans) | AR | rs104893843 | NM_000406.3 | c.30T>A | N10K (p.Asn10Lys) | missense | Likely Pathogenic | Likely Pathogenic | 0.000129 | 0.000229 |  |
| 344 | KS | no* | *GNRHR* | CH (trans) | AR | rs776834867 | NM_000406.3 | c.31C>A | Q11K (p.Gln11Lys) | missense | Likely Pathogenic | Uncertain significance | 0.000129 | 0.000229 |  |
| 344 | KS | no* | *GNRHR* | CH (trans) | AR | rs727505367 | NM_000406.3 | c.350T>G | L117R (p.Leu117Arg) | missense | Pathogenic | Likely Pathogenic | 0.0000131 | 0.0000294 |  |
